# Efficacy and safety of common Chinese herbal medicines in treating psoriasis: a systematic review and meta-analysis

**DOI:** 10.3389/fphar.2026.1718564

**Published:** 2026-02-20

**Authors:** Mengyun Zhou, Yang Sun, Tianhong Xu, Hui Zhang, Xiujiao Xia

**Affiliations:** 1 Department of Dermatology, Hangzhou Third People’s Hospital, Hangzhou Third Hospital Affiliated to Zhejiang Chinese Medical University, Hangzhou, China; 2 College of Pharmaceutical Science, Zhejiang University of Technology, Hangzhou, China

**Keywords:** effectiveness, herbal medicine, meta-analysis, psoriasis, traditional Chinese medicine

## Abstract

**Background:**

Psoriasis is a common chronic inflammatory skin disease with a high incidence, imposing a substantial global disease burden. As a low-cost complementary and alternative therapy, traditional Chinese herbal formulations have been widely used in clinical practice in China for the treatment of psoriasis. However, systematic analyses of their application value based on traditional Chinese medicine (TCM) syndrome patterns or treatment outcomes remain relatively limited.

**Objective:**

We aim to systematically assess the efficacy and safety of common traditional Chinese herbs in the treatment of patients with psoriasis.

**Methods:**

We conducted a systematic literature search in PubMed, Web of Science, SciFinder, WanFang Database, and China National Knowledge Infrastructure (CNKI). The literature meeting the predefined inclusion criteria was subjected to a secondary screening process. Meta-analysis was subsequently conducted using Review Manager software (version 5.4.1).

**Results:**

A total of 47 papers involving 3,675 patients were included in the study. All TCM (herbal formula) groups showed positive effects in enhancing the treatment efficiency of different types of psoriasis. In particular, herbal medicines had a more considerable role in managing arthropathic psoriasis compared to other types of psoriasis. In terms of reducing Psoriasis Area and Severity Index (PASI) scores, TCM formula treatment showed marked benefits in pustular psoriasis. As for the improvement in TCM symptom indicators, patients with erythrodermic psoriasis (EP) showed better efficacy. For psoriasis accompanied by blood dryness, herbal medicines were more effective in reducing Dermatology Life Quality Index (DLQI) scores.

**Conclusion:**

The results indicate that TCM formulations included in this review are beneficial for reducing PASI scores, DLQI scores, and TCM symptom scores among patients with different types of psoriasis. The most commonly used herbal formula was Blood cooling and detoxification formula, followed by Taohong Siwu decoction and Blood nourishing and detoxifying formula. Nevertheless, more rigorously designed studies on herbal medicines are warranted to enhance the overall quality of research in this field going forward.

## 1 Introduction

**FIGURE 1:**
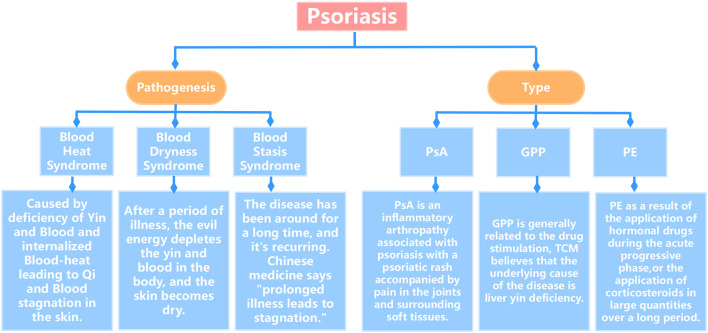
Classification chart of psoriasis.

Psoriasis is a common chronic inflammatory disease within the field of dermatology, characterized by its scales and erythematous plaques, which are commonly found on the scalp and limbs and even spread throughout the body (Raychaudhuri et al., 2014). According to statistics, the global prevalence of psoriasis fluctuates at approximately 2%–3%, affecting more than 125 million people worldwide (Armstrong and Read, 2020, 2020). This disease can occur at any age, with two-thirds of cases developing before the age of 40 (Committee on Psoriasis CSoD, 2023). The disease is characterized by high morbidity, long duration, and recurrent episodes. In biomedicine, psoriasis is primarily classified into the common, erythrodermic, arthritic, and pustular types. Correspondingly, traditional Chinese medicine (TCM) categorizes psoriasis into three patterns: blood-heat, blood-dryness, and blood-stasis (shown in Figure 1), in which the blood-heat type is the most common. Blood heat is embedded within the blood, resulting in poor Qi and blood circulation, along with stagnation of the skin. Clinically, the blood-heat type often manifests in the form of the rapid progression of rashes, with new rashes emerging continuously and existing rashes expanding. The rashes typically present as droplet-shaped lesions that are bright red in color and covered with layers of scales. Scraping off the scales reveals punctate bleeding. With the development of the disease, the blood-heat type may be transformed into blood-dryness type, which is caused by long-term illnesses depleting yin and blood and drying out the skin. Clinically, the blood-dryness type typically manifests as pale red, coin-shaped or large patches of skin, featuring less scaling and dry or cracked skin. Once the scaling is removed, there is usually no indication of new skin lesions. The blood stasis in psoriasis is frequently caused by prolonged heat toxicity and internal dryness, resulting in impaired blood circulation and insufficient nourishment of the skin with Qi and blood. The main clinical manifestations of the blood-stasis type include skin rashes, scales, and bleeding points that are exposed when the scales are removed from the affected skin areas. There is also cupping of the skin, stiffness of joints, thickening of skin lesions, a dark red hue, and persistence of the symptoms.

The systematic modern medical treatments for psoriasis, such as use of retinoids, immunosuppressants, and biologics, have shown some effectiveness. However, these treatments can have certain side effects and are often associated with high medical costs, causing substantial inconvenience to patients. TCM therapies, especially herbal formulas, play an important role in the treatment of psoriasis due to their unique advantages. The term “white sore” was first recorded in the book “Great Achievements in Surgery” in ancient China (Xiang, 2015). According to Chinese medicine theory, the development of psoriasis is closely related to the pathological processes of heat-toxin stagnation, loss of blood, loss of nourishment to the skin, and the development of dryness and wind. The modified Xijiao Dihuang decoction (Rhinoceros Horn and Rehmannia decoction), which was nominated and recommended in the “2017 Expert Consensus on TCM Treatment of Psoriasis,” is noted for its good application value in the treatment of patients with common psoriasis who have a blood-heat syndrome (Chendan Li et al., 2021). Professor Zhao Bingnan, a renowned dermatologist, created the Liangxue Huoxue decoction, adhering to the principle of treating diseases from the perspective of blood circulation. This provides more references and insights for the treatment of psoriasis (Chendan Li et al., 2021).

Nevertheless, there is a paucity of meta-analyses on clinical studies concerning the use of Chinese herbal formulations for the treatment of erythrodermic, arthritic, and pustular psoriasis. This study systematically integrated and evaluated the efficacy of Chinese herbal formulations in treating different types of psoriasis and their impact on related outcome measures by means of meta-analysis, with the aim of providing more robust evidence-based medical support for the clinical management of psoriasis.

## 2 Methods

### 2.1 Data source and search strategy

The literature on the treatment of different types of psoriasis with TCM (herbal formula) published externally was searched from PubMed, Web of Science, SciFinfer, WanFang Database, and China National Knowledge Infrastructure (CNKI). The search was carried out from the establishment of the database to 1 June 2024, and the contents of the search terms are shown in Table 1. This review was conducted according to the Preferred Reporting Items for Systematic Reviews and Meta-Analyses guidelines. The protocol for this systematic review was prospectively registered on the International Prospective Register of Systematic Reviews (PROSPERO) with the registration number CRD420251268213.

**TABLE 1:** Complete search strategy.

Database	Platform/Interface	Date of search	Search strategy	The number of search results
PubMed	NCBI	1 June 2024	1. (Psoriasis) AND (“blood stasis”) AND (“Taohong Siwu decoction”) 2. (Psoriasis) AND (“blood cooling” OR “detoxification formula”) AND (“blood heat syndrome”) 3. (Psoriasis) AND (“blood dryness”) AND (“blood nourishing and detoxifying formula”) 4. (Chinese medicine erythrodermic psoriasis) 5. (Chinese medicine pustular psoriasis) 6. (Chinese medicine arthropathic psoriasis)	154
CNKI	China National Knowledge Infrastructure Knowledge Discovery Network Platform	1 June 2024	1. SU=(psoriasis) AND SU= (bloodstasis) AND SU=(Taohong siwu decoction) Ibid	3,187
WanFang	WanFang Data Knowledge Service Platform	June 1, 2024	1.S=(Taohong siwu decoction) AND S=(Blood stasis) AND S=(Psoriasis)	2,013
CQVIP	VIP Chinese Journal Platform	1 June 2024	1.T = “Taohong siwu decoction”) AND (K = “Blood stasis”) AND (S = “Psoriasis”)	2,898

### 2.2 Eligibility criteria

We included randomized controlled trials (RCTs) evaluating oral multiherbal TCM formulas used alone or in combination with standard Western medications. The inclusion criteria were as follows: (1) randomized controlled trials in which the observation group received TCM formulas, while the control group received placebo and other therapeutic drugs for psoriasis; (2) studies in which patients met the biomedicine psoriasis diagnostic standard or TCM identification standard; and (3) studies involving patients with progressive psoriasis.

The exclusion criteria were as follows: (1) non-clinical studies, such as animal experiments, and (2) studies involving patients with chronic diseases other than psoriasis.

### 2.3 Study screening and data extraction

To reduce bias, data were extracted using the “two-individual extraction method” by reading the full text of the literature and collecting basic information about it (first author, year of publication, sample size, age, treatment period, treatment measures, and outcome indicators).

### 2.4 Risk-of-bias assessment

The quality of the included literature that met the screening criteria was evaluated by the investigators with reference to the “Risk of Bias Assessment Tool” recommended by the Cochrane Collaboration. The following aspects were evaluated: (1) randomized control; (2) allocation concealment; (3) blinding or not; (4) data completeness; (5) selective reporting; (6) other biases.

### 2.5 Data synthesis and analysis

After categorizing and summarizing the data from all the literature, primarily including primary outcomes [improvement in the Psoriasis Area and Severity Index (PASI)and clinical efficacy rate], secondary outcomes [Dermatology Life Quality Index (DLQI), TCM symptom score, and adverse events],we will then input the data into Revman5.4.1 software for analysis. In accordance with the Cochrane Handbook for Systematic Reviews of Interventions (*Section 6.5.2.2*), the standard deviation (SD) was calculated or imputed using the recommended Cochrane formulae, where necessary.

### 2.6 Statistical methods

All data were processed using Revman5.4.1 software, and the heterogeneity test was conducted before analysis. If *I* ^2^>50%, then *P*< 0.1, suggesting that the included literature was heterogeneous and the random-effect model was chosen. If *I* ^2^≤50%, then *P*≥ 0.1, suggesting that the included literature was not heterogeneous and the fixed-effect model was chosen. Effectiveness and adverse reactions, among other binary outcome measures, are analyzed using binary variables. The effect sizes included relative risk (RR) and 95% confidence interval (95%CI). For indicators such as PASI and DLQI, which can take any value within a certain range, continuous variable analysis should be used. The effect sizes included standard mean difference (SMD) and 95%CI, and *P*< 0.05 indicated that the differences were statistically significant. The analyses were presented in forest plots, and risk of bias was assessed using funnel plots, with *P*-values <0.05 indicating significant publication bias.

$$RR=\frac{a/c}{b/d},$$

$$SMD=\frac{\left(M_{1}-M_{2}\right)}{\left(SD_{1}+SD_{2}\right)/2}.$$

Notations:

*a*: Number of occurrences in the experimental group

*b*: Number of occurrences in the control group

*c*: Total number of individuals in the experimental group

*d*: Total number of individuals in the control group

*M*_1:_Variance of the experimental group

*M*_2:_Variance of the control group

SD_1_: Standard deviation of the experimental group

SD_2_: Standard deviation of the control group

## 3 Results

### 3.1 Literature selection and study characteristics

**FIGURE 2:**
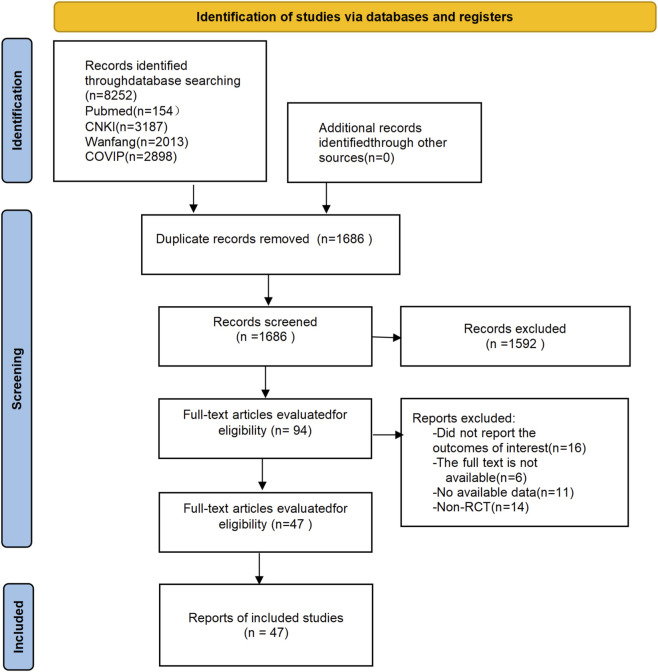
Overall flow chart of the literature analysis on psoriasis.

A flowchart of the selection process is presented in Figure 2. After searching for 8,252 citations, 94 full-text articles were assessed for eligibility. Forty-seven randomized controlled trials (3,675 adults) proved eligible according to the inclusion criteria. The sample sizes of the included trials ranged from 17 to 110 individuals. The duration of the intervention ranged from 4 to 12 weeks. The mean age was 39.41 years (standard deviation 5.2), and the mean duration of psoriasis was 8.47 years (standard deviation 6.46).

### 3.2 Characteristics of the included RCTs

All included studies (*n* = 47) that met the selection criteria were conducted in China and published in Chinese between 2007 and 2024. Among these studies, 47 RCTs were conducted in a single center. The detailed characteristics of the included RCTs can be found in Table 2.

**TABLE 2:** Basic characteristics of the included literature.

Inclusion of studies	Test group			Control subject			Course of treatment (weeks)	External medication
	Age	Sample size	Intervention	Age	Sample size	Intervention		
Yan, 2024	38.69 ± 4.87	40	Cooling blood and detoxifying formula + A vitamin A capsule + calcipotriol^a^	38.04 ± 4.35	40	A vitamin A capsule + calcipotriol	4	—
Zhou,2023	40.06 ± 2.23	35	Cooling blood and detoxifying formula + calcipotriol ointment^a^	44.2 ± 2.19	35	Calcipotriol ointment	8	Urea ointment
Wu, 2021	38.05 ± 7.89	40	Draining liver, cooling blood, and detoxifying formula^a^	38.23 ± 7.70	40	Compound indigo capsule^b^	8	Calcipotriol ointment
Hu, 2022	40	32	Cooling blood and detoxifying formula^a^	36	32	Diyin tablets^b^	8	Natural indigo ointment
Wang, 2020	36.28 ± 5.94	40	Cooling blood and detoxifying formula +Xiaoyin granules + calcipotriol ointment^a^	35.87 ± 5.87	40	Xiaoyin granules + calcipotriol ointment^b^	8	—
Hua and Liu, 2020	35.58 ± 5.43	53	Cooling blood and detoxifying formula + Tripterygium glycoside tablet^a^	34.73 ± 5.22	52	Tripterygium glycoside tablets^b^	8	Tacalcitol ointment
Wen, 2020	43.25 ± 14.64	32	Cooling blood and detoxifying formula^a^	43.10 ± 14.98	32	Diyin tablets^b^	8	Natural indigo ointment
Wei Shen et al., 2020	36 ± 14	31	^a^Cooling blood and detoxifying formula + calcipotriol ointment + mometasone furoate cream	35 ± 11	32	Calcipotriol ointment + mometasone furoate cream	8	Body lotion
Wang, 2019	40.05 ± 4.53	76	Cooling blood and detoxifying formula^a^	39.26 ± 4.79	76	Xiaoyin granules^b^	8	—
Hongbo Zhang and Zhang, 2019	53.3 ± 11.1	30	TCM decoction + compound glycyrrhizin tablets^a^	53.9 ± 10.0	30	Compound glycyrrhizin tablets	8	—
Changliang Mao et al., 2019	31.5 ± 15.72	48	Cooling blood and detoxifying formula + NB–UVB irradiation^a^	35.24 ± 12.35	48	NB-UVA light therapy	4	Vaseline
Liang, 2016	39.25 ± 11.74	34	^a^Clearing heat, cooling blood, and detoxifying decoction^a^	37.74 ± 10.28	34	Anthralin ointment	8	Anthralin ointment
Tang, 2012	38.29 ± 13.62	33	Cooling blood and strengthening spleen formula^a^	36.43 ± 11.55	33	Compound indigo capsule	8	—
Wei Li and Cai, 2011	35.83 ± 10.52	52	Cooling blood and detoxifying formula^a^	35.83 ± 10.52	53	Compound indigo capsule	8	Vaseline
Zhiyong Liu et al., 2010	34.59 ± 7.09	17	Cooling blood and detoxifying formula^a^	36.91 ± 11.52	23	Self-formulated prescription^a^	8	Vaseline or silicone cream
Wang, 2019	20∼ 65	50	Cooling blood and detoxifying formula^a^	22–50	30	Not have	8	—
Wen, 2023	37.23 ± 12.58	40	Modified Huoxue San Yu decoction^a^	36.85 ± 11.63	40	Yujin Yinxie tablets^b^	8	—
Jiamei Xiong et al., 2023	35.57 ± 9.78	30	Xiao Yin Tang + cupping therapy^a^	35.29 ± 9.63	30	Walking cupping therapy	8	Calcipotriol ointment
Hu, 2022	37.89 ± 10.01	50	^a^Taohong siwu decoction + A vitamin A capsules	41.45 ± 12.23	50	A vitamin A capsules	8	Tacrolimus ointment
Liu, 2022	38.22 ± 8.54	56	Blood circulation and detoxification formula + narrow-spectrum medium-wave ultraviolet rays^a^	36.95 ± 8.40	56	Narrow-spectrum medium-wave ultraviolet	8	—
Wu, 2021	38.29 ± 10.04	31	Modified Taohong Siwu decoction + herbal baths^a^	36.66 ± 10.28	29	^a^ Herbal bath	8	Body lotion
Ying Zhou et al., 2021	45.65 ± 8.33	40	Taohong siwu decoction + calcipotriol ointment^a^	45.32 ± 9.61	40	Calcipotriol ointment	8	—
Liu F. 2019	46.65 ± 9.33	43	Taohong Siwu Tang + compound glycyrrhizin injection^a^	46.32 ± 10.61	43	Compound glycyrrhizin injection	4	Psoriasis lotion topical + boric acid solution + trimethoprim acetate injection
Xuebing Zhang et al., 2017	30.1 ± 4.4	55	Taohong Ershao decoction + A vitamin A capsules^a^	29.8 ± 7.3	55	A vitamin A capsules	4	—
Wu , 2016	35.47 ± 12.31	32	NB–UVB irradiation +Taohong Siwu Tang^a^	34.32 ± 11.19	31	Xiaoyin granules^b^	8	Daivonex ointment
Wang, 2023	—	50	Qingre Yangxue Jiedu decoction + calcipotriol betamethasone ointment^a^	—	50	Calcipotriol betamethasone ointment	8	—
Yingjie Wang et al., 2022	33.25 ± 58.75	60	Yangxue Xuantou decoction^a^	35.25 ± 55.00	60	A vitamin A capsules	8	Urea ointment
Yingjie Wang et al., 2021	45.26 ± 12.30	46	Yangxue Xuantou decoction^a^	43.32 ± 12.65	46	^a^ Herbal bath	4	—
Tang, 2012	34.17 ± 1.75	42	Qingre Yangxue Jiedu decoction + calcipotriol betamethasone ointment^a^	33.25 ± 1.67	42	Calcipotriol betamethasone ointment	8	—
Suqing Yang and Wang, 2020	33.61 ± 9.12	45	Blood nourishing and emollient formula^a^	0.26 ± 9.00	45	Runzao Zhiyang capsule^b^	6	—
Liu D., 2019	39.23 ± 13.05	30	Modified Yangxue Jiedu decoction^a^	36.93 ± 12.40	30	Compound Zeqi granules^b^	8	—
Wang, 2020	58.5 ± 2.5	40	Nourish blood and resolve spot soup^a^	56.7 ± 2.1	40	Xiaoyin granules^b^	8	Vaseline
Chunyan Jiang et al., 2015	37.21 ± 9.87	78	Nourish blood and resolve spot soup^a^	35.86 ± 9.23	37	Xiaoyin granules^b^	8	Vaseline
Shi, 2017	40.6 ± 5.5	36	Nourish blood and resolve spot soup^a^	40.2 ± 5.3	35	Xiaoyin granules^b^	8	—
Liu, 2022	44.89 ± 10.67	35	Modified Jiedu Qingying decoction^a^	42.03 ± 9.53	33	Compound indigo capsule^b^	8	—
Lixin Sun and Shen, 2021	39.67 ± 4.28	30	Liangxue Xiaobi pills + A vitamin A capsules^a^	41.77 ± 3.96	30	A vitamin A capsules	6	—
Bao (2015)	41.26 ± 16.44	57	Modified Qingying decoction + A vitamin A capsule + compound glycyrrhizic acid injection^a^	42.46 ± 16.73	57	A vitamin A capsule + compound glycyrrhizic acid injection	8	—
Wei Li and Cai, 2011	42.35 ± 0.09	32	Modified Qingying Tang + A vitamin A capsule + compound glycyrrhizic acid injection^a^	41.20 ± 0.12	36	A vitamin A capsule + compound glycyrrhizic acid injection	4	—
Lihua Wang et al., 2009	38.8 ± 4.61	17	Qingre Huoxue formula + A vitamin A capsules^a^	37.5 ± 5.3	16	A vitamin A capsules	8	Poultice
Hongpu Yang, 2007	32.7 ± 8.21	40	Xin Yin ling granules + A vitamin A capsules^a^	33.1 ± 7.98	40	A vitamin A capsules	4	—
Jin, 2014	38.0 ± 2.4	25	Chinese herbal medicine^a^	39.0 ± 2.4	25	Compound glycyrrhizic acid injection	8	—
Fen Fu, 2020	46.6 ± 6.3	30	A vitamin A capsule + herbal baths^a^	46.4 ± 6.1	30	A vitamin A capsules	8	—
Guihua Liu, 2017	39.77 ± 6.65	30	Modified Longdan Xiegan decoction^a^	42.40 ± 7.03	30	A vitamin A capsules	8	Longzhu ointment^b^
Xianxing and Ban, 2014	18∼60	30	Yanghe decoction + A vitamin A capsules^a^	15∼58	30	A vitamin A capsules	8	—
Guan (2013)	42.25 ± 0.31	29	A vitamin A capsule + TCM^a^	42.25 ± 0.31	28	A vitamin A capsules	8	—
Geng (2013)	39.3	40	A vitamin A capsules + herbal baths^a^	39.3	40	A vitamin A capsules	8	—
Wei Luo, 2007	32.3	32	A vitamin A capsules + TCM^a^	31.2	36	A vitamin A capsules	3	—

^a^ Herbal formula based on pattern differentiation,

^b^ Chinese patent medicine. Based on the principle of syndrome differentiation and treatment (Bian Zheng Lun Zhi), the herbal components or dosages in each TCM formula may vary accordingly. The composition and processing of the herbal ingredients of compound preparation extracts and Chinese medicine formulas were verified using the ConPhYMP tool (https://ga-online.org/best-practice/). Please refer to the Supplementary Materials (S1–S6) for details.

### 3.3 TCM formulas and herbs of the included RCTs

Among the 47 studies using multi-ingredient TCM formulae, one formula named “Blood cooling and detoxification formula” was used in 16 studies. The main groups are*Rehmanniae Radix, Bubali Cornu, Sophorae Flos, Arnebiae Radix, Moutan Cortex, SalviaeMiltiorrhizae Radix Et Rhizoma, Hedyotis Diffusa, Paeoniae Radix Rubra,* and *Smilacis Glacis Rlabrae Rhizoma. Arnebiae Radix* is the most frequently used formula (*n* = 30), followed by *Rehmanniae Radix.* This formula is mainly used to treat blood-heat syndrome (Yan, 2024; Zhou, 2023; Sunsi Wu et al., 2022; Kaishu et al., 2023; Gang Wang, 2020; Hua and Liu, 2020; Wen, 2020; Wei Shen, 2020; Wang, 2019; Hongbo Zhang and Zhang, 2019; Changliang Mao et al., 2019; Liang, 2016; Tang, 2012; Zhihong Li, 2011; Zhiyong Liu et al., 2010; Lihua Wang et al., 2009). Among the 47 studies using multi-ingredient TCM formulae, one formula named “Taohong Siwu decoction” was used in eight studies. The main groups are*Persicae Semen, Carthami Flos, Paeoniae Radix Alba, Angelicae Sinensis Radix, Salviae Miltiorrhizae Radix Et Rhizoma,* and *Chuanxiong Rhizoma*. *Carthami Flos* is the most frequently used formula (*n* = 30), followed by *Persicae Semen.* This formula is mainly used to treat blood-stasis syndrome (Wen, 2023; Jiamei Xiong et al., 2023; Hu, 2022; Xiuling Liu and Chengbin, 2022; Wu, 2021; Ying Zhou et al., 2021; Liu F., 2019; Xuebing Zhang et al., 2017; Wu, 2016). In 47 studies using multi-ingredient TCM formulations, one formula named “Blood nourishing and detoxifying formula” was used in nine studies. The main groups are*Rehmanniae Radix, Angelicae Sinensis Radix, Smilacis Glacis Rlabrae Rhizoma, SpatholobCaulis, Salviae Miltiorrhizae Et Rhizoma,* and *Paeoniae Radix Alba. Angelicae Sinensis Radix* is the most frequently used formula (*n* = 30), followed by *Rehmanniae Radix.* This formula is mainly used to treat blood-dryness syndrome (Wang, 2023; Yingjie Wang et al., 2022; Yingjie Wang et al., 2021; Juan, 2021; Suqing Yang and Wang, 2020; Liu D., 2019; Chunyan Jiang et al., 2015; Chunyan Jiang et al., 2015; Shi, 2017).

**FIGURE 3:**
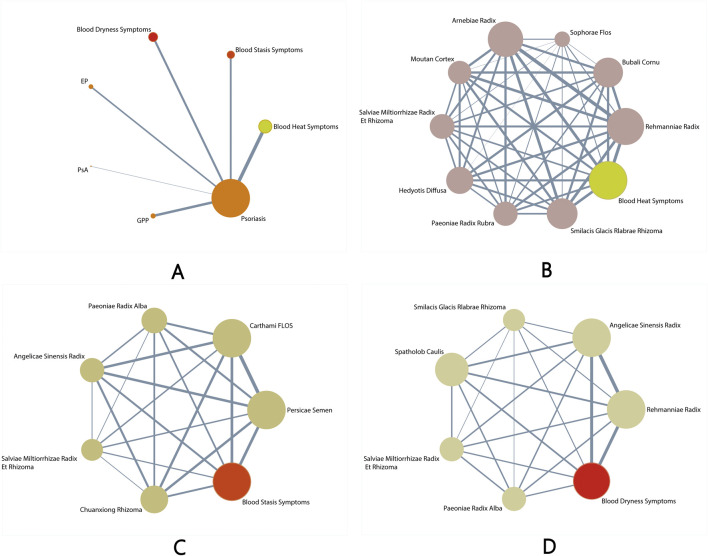
Available comparative networks. **(A)** The effectiveness of herbal remedies and placebo for different types of psoriasis. **(B)** Different single herbs and placebo for blood heat psoriasis. **(C)** Different single herbs and placebo on psoriasis with blood stasis. **(D)** Different single herbs and placebo for psoriasis with blood dryness.

Among all studies, a total of 116 herbs with the frequency of 200 were prescribed, with *Rehmanniae Radix* being the most frequently used herb (*n* = 30), followed by *Smilacis Glacis Rlabrae Rhizoma* (*n* = 19) and *Paeoniae Radix Rubra* (*n* = 16) (Figure 3).

### 3.4 Risk-of-bias assessment

**FIGURE 4:**
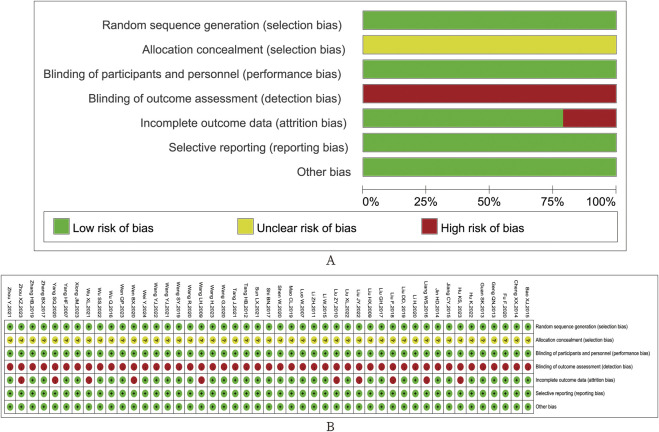
Risk of bias of the included studies. **(A)** risk-of-bias summary; **(B)** risk-of-bias traffic light.

Among all studies, 47 studies had a low risk of randomized sequence generation bias; all studies had an allocation scheme that was hidden and not mentioned in the text. All studies had a low risk of deviating from the intended intervention, 37 studies had a low risk of missing endpoint data, and 10 studies had incomplete endpoint data, but all accounted for withdrawn cases. There was a risk of follow-up bias of approximately 25%, and the endpoint domains in all studies measurements were at a low risk. The risk of selection reporting bias was low in all studies. Overall, the quality of the included RCTs was good (Figure 4).

### 3.5 Clinical efficacy

**FIGURE 5:**
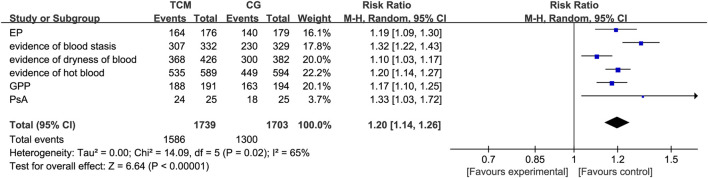
Meta-analysis for clinical efficacy at the end of treatment. CG, control group.

In studies assessing the efficacy of herbal remedies against different types of psoriasis, an in-depth analysis was carried out through the metric of the efficacy rate. The results show that compared to the control group, the TCM (herbal formula) group has a marked advantage in improving the efficacy of all types of psoriasis (RR: 1.20; 95% CI :1.14–1.26). This is especially evident in the case of arthropathic psoriasis, where TCM (herbal formula) treatment shows more pronounced improvements in clinical outcomes (RR: 1.33; 95% CI:1.03–1.72). The results of these analyses provide strong evidence to support the use of herbal formulas in the treatment of psoriasis (Figure 5).

### 3.6 Psoriasis area and severity index

**FIGURE 6:**
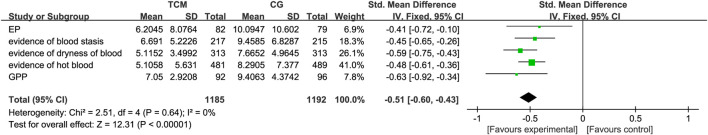
Meta-analysis for PASI at the end of treatment. CG, control group.

In the analyses addressing changes in PASI scores, a total of 31 clinical trials involving data from 2,377 participants were included in this study. The PASI is a standardized method used to assess the extent and severity of psoriasis, widely applied in clinical trials and research. The PASI score considers the area affected by lesions and the severity of the lesions, with higher scores indicating more severe conditions. The results showed that compared to the control group, the TCM (herbal formula) experimental group exhibited a marked reduction in PASI scores across all types of psoriasis (SMD: −0.51; 95% CI:−0.60 to−0.43). In particular, compared to other types of psoriasis, the TCM (herbal formula) experimental group demonstrated an even more significant effect in reducing the PASI scores of pustular psoriasis (SMD:−0.63; 95% CI:−0.92 to−0.34) (Figure 6).

### 3.7 Dermatology life quality index

**FIGURE 7:**
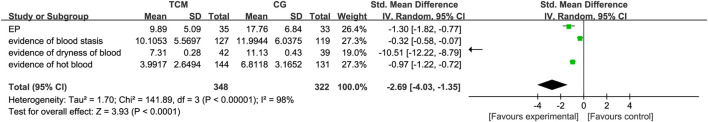
Meta-analysis for DLQI at the end of treatment. CG, control group.

For the analysis of changes in DLQI scores, we conducted nine trials with a total of 670 patients participating. DLQI is used to measure the impact of psoriasis on patients’ quality of life, with higher scores indicating a greater impact of the skin disease on the patient’s quality of life. The results show that, compared to the control group, the TCM (herbal formula) experimental group exhibited a lower DLQI score for all types of psoriasis patients (SMD: −2.69; 95% CI:−4.03 to−1.35). Especially when compared to other types of psoriasis, the TCM (herbal formula) experimental group showed a more significant effect in reducing the DLQI scores for patients with blood dryness psoriasis syndrome (SMD: −10.51; 95% CI:−12.22 to−8.79) (Figure 7).

### 3.8 TCM symptom scores

**FIGURE 8:**
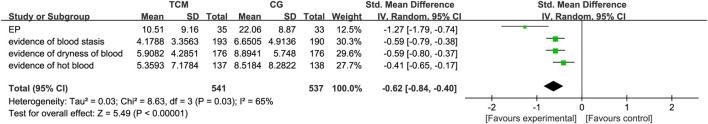
Meta-analysis for TCM symptom scores at the end of treatment.CG, control group.

In the analysis of TCM symptom scores, 14 clinical trials were involved, with a total of 1,078 patients participating in the observation of changes in scores. The TCM symptom score is a method used to evaluate the severity and changes in symptoms in patients, with higher scores indicating more severe symptoms. The results show that, compared to the control group, the TCM (herbal formula) experimental group exhibited a lower TCM symptom score for all types of psoriasis patients (SMD: −0.62; 95% CI:−0.84 to−0.40). Especially when compared to other types of psoriasis, the TCM formula group demonstrated more significant efficacy in reducing the TCM syndromic scores of erythrodermic psoriasis (EP) (SMD: −1.29; 95% CI:−1.79 to−0.74). On the other hand, TCM formula was relatively less effective in reducing TCM symptom scores of patients with blood-heat psoriasis (SMD: −0.41; 95% CI:−0.65 to−0.17). These data reflect differences in the efficacy of herbal formulas in the treatment of different types of psoriasis (Figure 8).

### 3.9 The effect of TCM in reducing adverse effects

**FIGURE 9:**
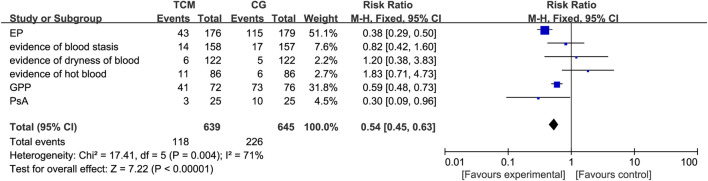
Meta-analysis based on adverse effects at the end of treatment for different types of psoriasis.CG, control group.

Data from a total of 1,284 participants were analyzed based on 17 publications documenting adverse events, which showed a statistically significant difference in adverse event reporting between the intervention and control groups. According to the comprehensive results of various studies, the TCM (herbal formula) experimental group showed a marked reduction in adverse reactions compared to the control group, implying a lower safety concern (RR: 0.54; 95% CI:0.45–0.63). However, for blood-heat psoriasis, blood-stasis psoriasis, and blood-dryness psoriasis, 95% CI is placed at the line of no-effect and is not statistically significant. The main reason may be that the number of patients experiencing adverse reactions in the TCM (herbal formula) experimental group was similar to that in the control group, and the weight of patients experiencing adverse reactions within the entire study was relatively low. This indirectly indicates that the TCM (herbal formula) experimental group had markedly reduced occurrences of adverse reactions than the control group. Among the different types of psoriasis, arthropathic psoriasis showed a more significant safety advantage[RR: 0.03; 95% CI:0.09–0.96). With regard to adverse reactions, minor symptoms such as itchy skin, gastrointestinal discomfort, and dry mouth and vomiting were more frequently reported, and these usually resolved on their own without specific medical intervention (Figure 9).

### 3.10 Subgroup analysis of treatment cycle times

**FIGURE 10:**
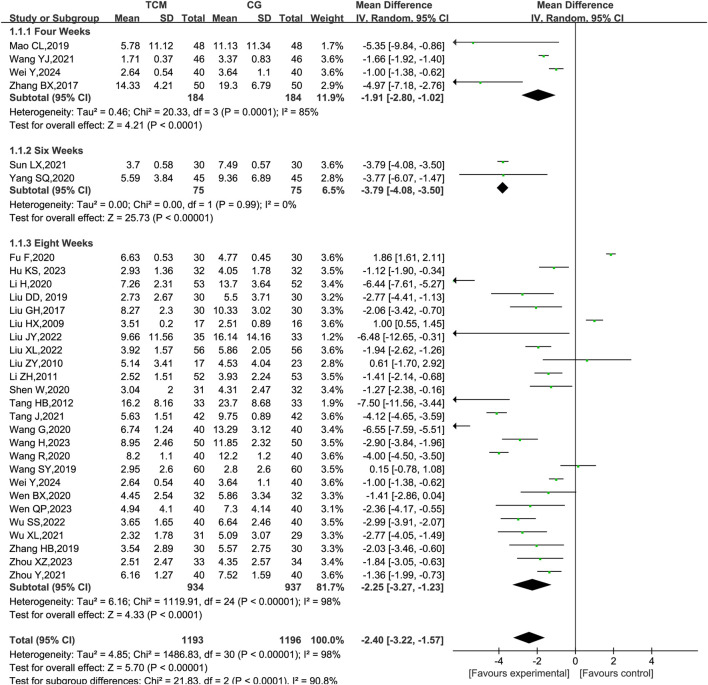
Subgroup meta-analysis based on PASI at the end of treatment for different treatment cycles. CG, control group. Note (Figures 5–10): The size of the nodes is proportional to the number of trial participants, and the thickness of the line connecting the nodes is proportional to the number of participants in randomized trials that directly compared the two types or TCM. These numbers represent the number of trials that contributed to each treatment comparison.

The efficacy of herbal remedies may be affected by the duration of the treatment cycle. To explore this issue in depth, we subdivided the treatment cycle into 4, 6, and 8 weeks and conducted subgroup analyses. The results indicated that the reduction in PASI scores by herbal remedies exhibited a relatively stable trend across different follow-up times. In particular, the reduction in PASI scores owing to herbal remedies was most significant at a treatment cycle of 6 weeks (SMD: −3.79; 95% CI:−4.08 to−3.05). In contrast, psoriasis patients with a treatment period of 8 weeks exhibited a slightly lower reduction in PASI scores (SMD: −2.25; 95% CI:−3.27 to−1.23) than those with a treatment period of 4 weeks (SMD: −1.91; 95% CI:−2.80 to −1.02). These findings contribute to a more comprehensive understanding of the changes in the efficacy of herbal formulas across treatment cycles (Figure 10).

## 4 Discussion

In ancient China, psoriasis was called “White sore,” which was first observed in “Great Achievements in Surgery”: “It is rheumatism and evil qi, which are born from the incarnation of the intestines, the regeneration of cold and dampness, and the struggle with blood and qi.” According to the theory in The Yellow Emperor’s Inner Canon (HuangdiNeijing), which postulates that “internal manifestations are necessarily reflected externally,” psoriasis is considered an internal disharmony of Qi and blood manifesting on the skin surface. The *Yizong Jinjian* (*Golden Mirror of the Medical Tradition*) describes psoriasis as follows: “It manifests on the skin as papules or crusts, white in color and itchy. When scratched, white scales detach. It is caused by pathogenic wind invading the skin surface, leading to blood depletion which fails to nourish the skin adequately.” The pathogenesis of psoriasis is closely associated with such internal disharmony. Through internal herbal medicine and other modalities, TCM aims to regulate the internal organs, meridians, and the balance of Qi and blood. This fundamental harmonization of the body’s internal environment targets the root cause, with the goal of eliminating lesions, achieving cure, and reducing recurrence.

Regarding psoriatic conditions, the findings of this study demonstrate that TCM formulations included in this review can yield considerable clinical benefits: markedly improving psoriatic arthritis; substantially reducing the PASI score in pustular psoriasis; markedly lowering the DLQI score in psoriasis with blood-dryness syndrome; and notably decreasing the TCM symptom score in EP.

TCM treatment emphasizes a holistic approach. For psoriasis patterns stemming from blood heat, blood stasis, or blood dryness, the application of herbs that clear heat, activate blood circulation, and nourish blood can not only alleviate surface symptoms such as erythema and scaling but also regulate systemic blood circulation and improve visceral function. This comprehensive strategy addresses the root internal factors, thereby reducing the likelihood of recurrence.

The results of this study indicate that the different Chinese herbal formulas all contain herbs for tonifying Qi and blood. Through modification (adding or subtracting herbs) based on specific symptoms, these formulae are enabled to clear heat and cool blood, activate blood circulation and resolve stasis, or nourish blood and stop bleeding. Analysis of the herb frequency revealed that within blood-cooling and detoxifying formulae, *Arnebiae Radix* was the most frequently used herb, followed by *Rehmanniae Radix*. In the Taohong Siwu decoction, *Carthami Flos* was the most common herb, followed by *Persicae Semen*. For blood-nourishing and detoxifying formulae, *Angelicae Sinensis Radix* was used most frequently, followed again by *Rehmanniae Radix*. We provide a rationale for selecting Chinese herbal formulas for different psoriatic syndrome types.

The treatment of TCM is based on the principle of differentiated diagnosis and treatment, and individualized regimens are provided in accordance with the specific circumstances of each patient. For psoriasis with blood heat, herbal formulas for cooling blood and clearing heat can be utilized. For psoriasis with blood deficiency and wind dryness, herbs for nourishing blood and moistening can be employed. This individualized treatment plan can more precisely target the patient’s condition and enhance the treatment efficacy. TCM medicine holds unique advantages in alleviating the symptoms of psoriasis. Some herbs with anti-itching and moisturizing effects can effectively relieve the patient’s skin itching symptoms and make the patient feel at ease. As TCM is primarily derived from natural plants, animals, and minerals, it is generally less costly than biological agents. The results indicated that the herbal medicine treatment group demonstrated a considerable advantage in terms of adverse effects, which was particularly pronounced in cases of psoriatic arthritis. However, the potential side effects associated with the combined use of multiple TCM or the integration of TCM and biomedicine warrant careful attention.

Based on the method of cooling blood and detoxifying as the principle for treating psoriasis with blood-heat syndrome, the comprehensive treatment of TCM with Liangxue Jiedu decoction as the core recipe has a definite clinical effect for psoriasis (Li-jun et al., 2021). The cooling blood and detoxifying decoction is derived from the basic formula of cooling blood and activating blood circulation, and all the herbs in the formula possess excellent effects of cooling blood, activating blood circulation, and clearing heat and detoxifying. Among them, *Poria cocos* has remarkable effects in eliminating dampness and detoxifying; *Rehmannia root* and *Angelica root* have outstanding effects of cooling blood, clearing heat, and nourishing yin; *White Peony* has excellent effects of cooling blood, clearing heat, activating blood circulation, unblocking meridians, nourishing yin, and moistening the tongue and lips; *Scrophularia ningpoensis* and *Fresh Gelsemium* have favorable effects of nourishing the stomach, generating saliva, nourishing yin and moistening lungs, and clearing the heart and relieving anxiety; *Phellodendron amurense*, *Gardenia fruit*, and *P.cocos* have good efficacy in clearing heat and dampness, eliminating fire and anxiety, and cooling blood; *P.cocos* and *White Swallowwort* have positive effects of detoxifying, clearing heat, and eliminating dampness. When these herbs are used in combination, they will enhance the effect of clearing heat and detoxifying without harming yin. In addition, they also have remarkable effects of stopping itching and eliminating dampness, which is a reliable approach to treating the blood-heat type of common psoriasis (Jun and Ying, 2018).

Taohong Siwu decoction can effectively improve the symptoms of Qi stagnation and blood-stasis psoriasis, demonstrating marked clinical efficacy, a low recurrence rate, and a favorable safety profile (Hu K, 2022). Taohong Siwutang demonstrates a good anti-inflammatory effect and skin barrier repair function in the treatment of psoriasis. It can considerably reduce the levels of inflammatory mediators in the peripheral blood and skin lesions and promote the expression of caspase-14 and recombinant envoplakin involved in skin barrier repair in the mouse model of psoriasis (NIU et al., 2024). Yangxue Jiedu decoction combined with TCM bath has a marked clinical effect on psoriasis of blood-dryness syndrome, which can improve the skin lesion area, the severity of skin lesions, the symptoms of itching, and the quality of life of patients (Jing-jing et al., 2021).

Over the past 2 decades, considerable advances have been made in the treatment of psoriasis, particularly with the development of highly effective biologic agents. Both biologics and small-molecule therapies are now available for patients with moderate-to-severe disease. IL-17 pathway inhibitors remain a cornerstone of treatment, with next-generation agents targeting IL-17A or IL-17F showing enhanced efficacy. For example, sonelokimab, a nanobody against IL-17A/IL-17F, and izokibep, a small-molecule IL-17A inhibitor, have demonstrated high PASI and ACR response rates, underscoring the therapeutic potential of small molecules in psoriasis (Mastorino L et al., 2025). In parallel, inhibition of the JAK-STAT pathway—especially through TYK2—has emerged as a promising therapeutic strategy in psoriatic arthritis (PsA). Selective TYK2 inhibitors such as deucravacitinib and zasocitinib have shown robust efficacy in psoriasis (Armstrong AW et al., 2024). Meanwhile, growing attention is being paid to the role of the gut microbiome in immune-mediated and autoimmune diseases. Novel interventions aimed at modulating the microbiome, including fecal microbiota transplantation (FMT), as well as prebiotic and probiotic supplements, are being explored as potential treatments for moderate-to-severe plaque psoriasis (Yang R et al., 2023).

## 5 Conclusion

We found that TCM formulations included in this review are beneficial for reducing PASI scores, DLQI scores, and TCM symptom scores among patients with different types of psoriasis. The most commonly used herbal formula was blood cooling and detoxification formula, followed by Taohong Siwu decoction and blood nourishing and detoxifying formula. However, further investigation is needed into the mechanisms of action and standardized treatment protocols of TCM herbal medicine in psoriasis management, while its potential for synergistic use with biologics and small-molecule targeted agents offers promising prospects for future research.
