# Supplementary material for: Efficacy and safety of common Chinese herbal medicines in treating psoriasis: a systematic review and meta-analysis
Source: Front Pharmacol. 2026 Feb 20;17:1718564. doi: 10.3389/fphar.2026.1718564 (PMC12964141; doi:10.3389/fphar.2026.1718564)
Supplement: Supplementary file 3 [file Supplementaryfile1.docx]

|  | Drug name | Composition of Traditional Chinese medicine | Description of the extract and extraction process | Preferred methods for  chemical analysis | References |
| --- | --- | --- | --- | --- | --- |
| **Chinese Herbal Formula** | Cooling Blood and Detoxifying Formula | Moutan Cortex, Phellodendri Cortex, Polygoni Cuspidati Rhizoma et Radix, Sanguisorbae Radix each 20 g, add 500 mL water. | Bring to a boil over high heat, simmer until reduced to 100 mL, cool for use. | / | (Huang Leijuan 2023) |
|  | Draining Liver, Cooling Blood and Detoxifying Formula | Moutan Cortex, Paeoniae Radix Rubra each 9 g; Isatidis Folium, Arnebiae Radix seu Lithospermi Radix each 15 g; Salviae Miltiorrhizae Radix et Rhizoma, Smilacis Glabrae Rhizoma, Bubali Cornu, Rehmanniae Radix Crudus, Hedyotis Diffusae Herba, Imperatae Rhizoma each 30 g. | Decoct in water and take warm. | / | (Wu Sunsi 2022) |
|  | Clearing Heat, Cooling Blood and Detoxifying Decoction | Bistortae Rhizoma 9g; Arnebiae Radix seu Lithospermi Radix, Paeoniae Radix Rubra, Dictamni Cortex each 10g; Lonicerae Japonicae Flos 12g; Sophorae Flos Immaturus, Smilacis Glabrae Rhizoma, Imperatae Rhizoma, Rehmanniae Radix Crudus each 25g. | Routine decoction. | / | (Wenqing. 2020) |
|  | Cooling Blood and Strengthening Spleen Formula | Crataegi Fructus, Sophorae Flavescentis Radix each 15g; Rehmanniae Radix Recens, Sophorae Flos Immaturus, Lonicerae Japonicae Flos, Alismatis Rhizoma each 20g; Bubali Cornu, Astragali Radix Crudus, Paeoniae Radix Rubra, Paris Polyphyllae Rhizoma, Dioscoreae Rhizoma, Coicis Semen Crudus each 30g. | One dose every two days, 200ml each time. | / | (Haobo 2012) |
|  | Modified Huoxue San Yu Decoction | Sparganii Rhizoma, Curcumae Rhizoma, Sappan Lignum, Euonymi Ramulus, Aucklandiae Radix, Tribuli Fructus, Scutellariae Radix each 10g; Paeoniae Radix Rubra, Paeoniae Radix Alba, Persicae Semen, Carthami Flos, Saposhnikoviae Radix, Dictamni Cortex, Lonicerae Japonicae Flos, Forsythiae Fructus, Citri Reticulatae Pericarpium each 15g; Coicis Semen Tostum 20g. | Decoct and take warm. | / | (Qiuping 2023) |
|  | Xiao Yin Tang | Rehmanniae Radix, Imperatae Rhizoma, Paeoniae Radix Rubra：10 parts each；Smilacis Glabrae Rhizoma, Dictamni Cortex, Anemarrhenae Rhizoma12 parts each；Lonicerae Flos，Saposhnikoviae Radix：6 parts each；Salviae Miltiorrhizae Radix et Rhizoma 15 parts,Glycyrrhizae Radix et Rhizoma 5 parts | Combine the crude drugs, decoct twice with water (first decoction 25–30 min, second 10–15 min), mix the two decoctions, and use the combined liquid. | / | Patent: CN102100862A |
|  | Taohong Siwu Decoction | wine-processed Angelicae Sinensis Radix, Rehmanniae Radix Praeparata, stir-fried Paeoniae Radix Alba：10 parts each；  Chuanxiong Rhizoma, soaked Persicae Semen：6 parts each；  Carthami Flos: 4 parts | Place the above crude drugs in a decocting apparatus, soak, then perform first and second decoctions; mix the two decoctions uniformly. | HPLC：octadecylsilyl-bonded silica gel; A = acetonitrile, B = 0.05–0.3 % phosphoric acid; gradient elution at 0.8–1.2 mL min^-1^; column temperature 25–35 °C; detection wavelength 210–400 nm. | Patent: CN114949074A |
|  | Modified Taohong Siwu Decoction | Persicae Semen, Chuanxiong Rhizoma, Paeoniae Radix Alba, Angelicae Sinensis Radix, Smilacis Glabrae Rhizoma, Smilacis Chinae Rhizoma：10 g each ；  Carthami Flos, Spatholobi Caulis, Clematidis Radix et Rhizoma, Salviae Miltiorrhizae Radix et Rhizoma：15 g each | Decoct one bag with water twice daily; combine the two decoctions to obtain 200 mL of final liquid for each administration. | / | (Xuling 2021) |
|  | Huoxue Jiedu Decoction | Smilacis Glabrae Rhizoma：30 g  Sophorae Tonkinensis Radix, Glycyrrhizae Radix et Rhizoma：6 g each  Arnebiae/Lithospermi Radix, Rehmanniae Radix, Moutan Cortex, Scrophulariae Radix, Angelicae Sinensis Radix：10 g each  Hedyotidis Diffusae Herba, Salviae Miltiorrhizae Radix et Rhizoma：15 g each | Place the weighed crude drugs in a round-bottom flask, add 10 volumes of distilled water, soak 30 min, decoct gently for 2 h, and filter; add 8 volumes of water to the residue, decoct again for 2 h, filter, and combine the two filtrates. | UPLC：Waters ACQUITY UPLC BEH C18 (100 mm × 2.1 mm, 1.8 µm); column temp. 40 °C; mobile phase A = 0.1 % formic acid in water, B = 0.1 % formic acid in acetonitrile; flow 0.3 mL min^-1^; injection 5 µL.  Gradient: 0 min 98 % A; 0–14 min 98→40 % A; 14.0–16.5 min 40→2 % A; 16.5–20.0 min 2 % A. | (Zhang Xiaolan 2025)  (Yang Xinxin 2020) |
|  | Taohong Ershao Decoction | Persicae Semen, Paeoniae Radix Rubra, Paeoniae Radix Alba, Curcumae Rhizoma, Salviae Miltiorrhizae Radix et Rhizoma, Spatholobi Caulis, Glycyrrhizae Radix et Rhizoma each 15 g  Carthami Flos, Chuanxiong Rhizomaeach 20 g  Sparganii Rhizoma, Euonymi Alatus Ramulus, Rumex madaio Mak., Rehmanniae Radix (raw), Sophorae Flos each 10 g | Decoct one daily dose with water and concentrate to 150 mL. | / | (Zhang Xuebing 2017) |
|  | Modified Jiedu Qingying Decoction | Taraxacum mongolicum, Smilax glabra rhizome 30g each, Paeonia veitchii root, Lonicera japonica flower, Imperata cylindrica rhizome, Forsythia suspensa, Moutan cortex, Rehmannia glutinosa, Tribulus terrestris 20g each, Saposhnikovia divaricata root, Phaseolus radiatus coat, Rubia cordifolia root, Ligusticum chuanxiong rhizome, Angelica sinensis, Gardenia jasminoides fruit 15g each, Coptis chinensis rhizome 10g. | Decoct most of the herbs with water multiple times, each time for 1 hours. Combine the decoctions and filter. For herbs containing liposoluble components, ethanol extraction can be used to improve the extraction yield of active components. Concentrate the extract under reduced pressure to remove excess water, obtaining a thick extract. Add ethanol to the concentrated liquid to a certain concentration, let stand for alcohol precipitation, remove impurities, recover ethanol from the supernatant. Subject the purified liquid to low-temperature drying to obtain. | / | (Jiying 2022) |
|  | Liangxue Xiaobi Pills | Rehmannia glutinosa 15g, Arnebia euchroma/Lithospermum erythrorhizon 10g, Moutan cortex 10g, Salvia miltiorrhiza 15g, Polygonatum sibiricum 15g, Eclipta prostrata 10g, Dioscorea opposita 10g, Hedyotis diffusa 15g, Isatis indigotica leaf 15g, Scutellaria barbata 10g, Dictamnus dasycarpus bark 10g, Glycyrrhiza uralensis 6g | Packaged in aluminum-plastic blister packs, each pill weighs 9g. | / | (Sun Lixin 2021) |
|  | Qingre Huoxue Formula | Rehmannia glutinosa, Sanguisorba officinalis root, Isatis indigotica leaf, Isatis indigotica root, Spatholobus suberectus vine, Smilax glabra rhizome, Imperata cylindrica rhizome, each 30g; Salvia miltiorrhiza, Moutan cortex, Angelica sinensis, Paeonia lactiflora root (red), Paeonia lactiflora root (white), each 10g; Hedyotis diffusa 15g | Proportion the required herbal materials according to the prescription, with conventional dosages such as Paeonia lactiflora root (red), Moutan cortex, Salvia miltiorrhiza, each 9-15g. Soak the herbs in water for 30 minutes, bring to a boil over high heat, then decoct over low heat for 20-30 minutes. Filter and collect the decoction; the dregs can be decocted a second time. | / | (Liu Hongxia 2009) |
|  | Modified Longdan Xiegan Decoction | Gentiana scabra, Gardenia jasminoides fruit, Scutellaria baicalensis, Alisma orientale, Angelica sinensis, and Bupleurum chinense, 9g each;  Glycyrrhiza uralensis, 6g;  Lonicera japonica flower and Smilax glabra rhizome, 30g each;  Plantago asiatica seed (wrapped for decoction), Rehmannia glutinosa, and Moutan cortex, 15g each. | Decoct in water for oral administration. | / | (Liu Guihua 2017) |
|  | Yanghe Decoction | Prepared Rehmannia glutinosa 30g, Cervus nippon antler glue 9g, Cinnamomum cassia bark (superior) 3g, Glycyrrhiza uralensis 3g, Zingiber officinale rhizome (processed) 1.5g, Ephedra sinica 1.5g, Sinapis alba seed 6g | Clean and select each herb, accurately weigh each according to the optimal proportions, add 8-20 times the amount of water, soak for 2-12 hours, heat-extract 2-3 times, combine the extracts, collect the volatile oil; let the aqueous extract stand for 12-48 hours, filter and collect the supernatant, concentrate under reduced pressure to obtain an extract or spray dry to obtain dry powder as the aqueous extract. | / | (Cheng Xianxiang 2014) |
| **Chinese patent medicine** | Compound Indigo Capsule | Indigo Naturalis, Rhizoma Cyrtomii, Crataegi Fructus Tostus, Massa Medicata Fermentata (Jian Qu) each 60g; Radix Arnebiae seu Lithospermi, Herba Taraxaci each 80g; Radix et Rhizoma Salviae Militiorrhizae, Rhizoma Dioscoreae Hypoglaucae, Cortex Dictamni, Schisandrae Chinensis Fructus (Alcoholic), Radix Angelicae Dahuricae each 100g; Mume Fructus, Herba Portulacae, Rhizoma Smilacis Glabrae each 200g. | / | Column: Dalian Elite Hypersil ODS (250mm×4.6mm, 5 μm). Mobile phase: Chloroform-methanol-0.1% acetic acid aqueous solution (6:59:35). Flow rate: 1.0 mL/min. | [https://pharma.bcpmdata.com/](https://pharma.bcpmdata.com/); (Dai Yuntao 2007) |
|  | Urea Ointment | Urea, Adeps Lanae each 100g; Cera Flava 40g; Glycerin 200g; Vaselinum quantum sufficiat to make 1000g. | Beeswax, lanolin, and yellow vaseline are melted in a water bath. Separately, urea and glycerin are heated in a warm water bath to dissolve urea, then slowly added to the base cooled to 50°C with constant stirring until cooled. | TLC:Using silica gel G plates with a mobile phase of absolute ethanol and 13.5 mol/L ammonia solution (99:1).  UV-Vis spectrophotometry :measure absorbance at 420 nm | (Su Huanhuan 2017)  Chinese Pharmacopoeia Commission. Chinese Pharmacopoeia 2020 Edition Online Database [DB/OL]. [2025.12.27].  [https://ydz.chp.org.cn/](https://ydz.chp.org.cn/). |
|  | Calcipotriol Ointment | Vitamin D3 derivative | / | HPLC: Isocratic elution. Mobile phase includes A (water) and B (acetonitrile) with a volume ratio of 45:55. Column packing: Octadecylsilane-bonded silica gel. Column length: 50-300 mm; Diameter: 1-10 mm; Particle size: 1-10 μm. Flow rate: 0.5-1.5 mL/min. Injection volume: 10-50 μL. Column temperature: 20-40°C. UV detector wavelength: 200-300 nm. | CN202110798115 |
|  | Xiaoyin Granules | Rehmannia glutinosa, Moutan bark, Paeonia lactiflora (red), Angelica sinensis, Sophora flavescens, Honeysuckle, Scrophularia ningpoensis, Arctium lappa, Cicada slough, Dictamnus dasycarpus, Saposhnikovia divaricata, Isatis indigotica leaf | Prepared into granules using conventional granule preparation methods. | HPLC : Lichrospher - C18 (250 mm × 4.6 mm, 5 μm). Mobile phase: Acetonitrile - 0.4% phosphoric acid aqueous solution (10:90, V/V). Flow rate: 1.0 mL/min. Detection wavelength: 327 nm. Column temperature: 30°C. Injection volume: 10 μL. | (Jiang Jingyi 2022)  CN202410380438 |
|  | Mometasone Furoate Cream | Single-Component | / | HPLC：Amethyst C8 (5 μm, 150 mm × 4.6 mm);  Mobile Phase: Methanol - Water (72:28, v/v);  Injection Volume: 10μL;  Wavelength: 254 nm;  Flow Rate: 1.0 mL·min^-1^ | (Li Wei 2014) |
|  | Tacalcitol Ointment | Single-component, active vitamin D₃ derivative | / | An octadecylsilyl-bonded silica gel column was used as the stationary phase with an acetonitrile–water mixture as the mobile phase; notably, the test and reference solutions were prepared by a sequential vortex-extraction-concentration protocol. | CN108663441A  (Zhang Hengpo 2017) |
|  | Tripterygium Glycosides Tablets | Tripterygium wilfordii 85–95 parts, traditional Chinese medicine excipient 5–10 parts, magnesium stearate 0.5–5 parts, maltodextrin 5–10 parts.  The traditional Chinese medicine excipient is a mixture of Lysimachiae Herba (Jinqiancao), Glycyrrhizae Radix et Rhizoma (Gancao), and Scutellariae Radix (Huangqin) in a mass ratio of (1–3):(1–3):(1–3). | First, a blend of Lysimachiae Herba, Glycyrrhizae Radix et Rhizoma, and Scutellariae Radix is pulverized, macerated in ethanol, and subjected to heat-reflux extraction. The resulting filtrate is concentrated under reduced pressure to produce a herbal extract. Next, Tripterygium wilfordii is mixed with neutral alumina, ground, and loaded onto a silica-gel column for purification through sequential soaking and elution with chloroform and a specified organic solvent mixture. The collected eluate is concentrated to obtain solid tripterygium glycosides. Finally, this purified extract is uniformly blended with the herbal extract, magnesium stearate, and maltodextrin, then dried and compressed into tablets. | TLC: use wilforlide A as the reference standard, cyclohexane–acetone (5:3) as the mobile phase, visualize with 10 % ethanolic sulfuric acid, and heat at 105 °C.  HPLC: Diomonisl C18 analytical column; detection wavelength 218 nm; column temperature 35 °C; mobile phase water–acetonitrile; flow rate 1.0 mL min-1; gradient elution. | CN106038661A  (Xiong Zhihui 2017)  (Yang Chunxin)  (Zhongshan Hospital 2008) |
|  | Compound Glycyrrhizin Tablets | Monoammonium glycyrrhizinate 10–15 parts, glycine 10–15 parts, DL-methionine 10–15 parts, disintegrant 5–20 parts, binder 0.2–5 parts, lubricant 0.2–5 parts, glidant 0.2–5 parts, diluent 5–40 parts, coating powder 0.5–5 parts. | All solid raw materials are sieved through a 100-mesh screen.Glycyrrhizinate, glycine, methionine, and excipients are pre-mixed and granulated via dry granulation using a roller compactor.The granules are uniformly blended with the glidant and the remaining lubricant.The contents of glycyrrhizinic acid, glycine, and methionine in the intermediate granules are determined by HPLC and must meet internal specifications.Qualified granules are compressed into cores based on the tablet weight calculated from the assay results.The tablet cores are film-coated by spraying an 8% (w/w) coating suspension in a coating pan to obtain the finished product. | HPLC:novel silica-based stationary phase; mobile phase acetonitrile–0.01 mol/L ammonium formate (85:15, v/v); flow 0.5–1.5 mL min^-1^; UV detection 200 nm; column temperature 20–40 °C; resolution between glycine and methionine ≥ 2.0; | CN106214656A |
|  | Medical White Petrolatum | Type: single-component excipient | semi-solid mixture of hydrocarbons obtained from petroleum and decolorized | Melt 2.0 g of the sample, add 2 ml of water and 0.2 ml of 0.05 mol/L iodine solution, shake, and cool; the upper layer shall be purple-pink or brown. | Chinese Pharmacopoeia Commission. Chinese Pharmacopoeia 2020 Online Database [DB/OL]. [2025-12-27]. |
|  | Yujin Yinxie Tablets | Radix Gentianae Macrophyllae, Radix Angelicae Sinensis , Rhizoma Acori Tatarinowii , Cortex Phellodendri Amurensis , Rhizoma Cyperi , Radix Curcumae, Rhizoma Curcumae Zedoariae, Realgar , Semen Strychni Powder , Spina Gleditsiae , Semen Persicae , Flos Carthami , Olibanum，30g each, Sal Ammoniac 12g, Natrii Sulfas Exsiccatus , Radix et Rhizoma Rhei 18g each, Eupolyphaga Steleophaga 36g, Indigo Naturalis, Semen Momordicae ，24g each | The above nineteen ingredients are ground into powder and soaked in 70% ethanol as the solvent for 24 hours, then percolated. The other six ingredients, including soapberry thorn, are boiled in water three times, with the first time for 3 hours, the second time for 2 hours, and the third time for 1 hour. The liquid is filtered, and the filtrate is combined and concentrated into a thick paste, which is then mixed evenly with the above thick paste and fine powder. Drying, crushing, sieving, granulation, drying, tablet pressing, sugar coating or film coating, and it is ready. | TLC:Prepare a reference solution containing 0.5 mg per mL of berberine hydrochloride in methanol. Develop using the upper layer of n-butanol-glacial acetic acid-water (7:1:2) as the mobile phase. Examine under UV light (365 nm).  HPLC:Use octadecylsilane bonded silica gel as the stationary phase. The mobile phase consists of acetonitrile-0.01 mol/L sodium heptanesulfonate mixed with 0.02 mol/L potassium dihydrogen phosphate in equal volumes (pH adjusted to 2.8 with 10% phosphoric acid) (21:79). Detection wavelength: 254 nm. | Chinese Pharmacopoeia Commission. Chinese Pharmacopoeia 2020 Online Database [DB/OL]. [2025.12.27]. |
|  | Tacrolimus Ointment |  | Dissolve an appropriate amount of tacrolimus in propylene carbonate, add it to the molten oil-soluble base , homogenize at 70°C for 15 min (3200 r·min^-1^), cool to room temperature to obtain the ointment, and store at ambient temperature. | HPLC:Zorbax SB C18 (2.1 mm × 150 mm, 3.5 µm); column temperature: 30°C; mobile phase: methanol (A) – 0.1% formic acid (containing 5 mmol·L^-1^ammonium acetate) (B); gradient elution: 0–0.5 min, 80% A; 0.5–5 min, 80% → 95% A; 5–7 min, 95% → 100% A, hold for 2 min; flow rate: 0.3 mL·min^-1^; injection volume: 10 µL; detection wavelength: 222 nm. | (Shan Ruiping 2022) |
|  | Acitretin Capsules | Acitretin (C_21_H_26_O_3_): Should contain not less than 90.0% and not more than 110.0% of the labeled amount. | / | / | Chinese Pharmacopoeia Commission. Chinese Pharmacopoeia 2020 Online Database [DB/OL]. [2025.12.27]. |
|  | Dithranol Ointment | Dithranol 0.1% Lactic acid 0.5%-1.5% Glycerin 40%-50% Hydroxypropyl methylcellulose aqueous solution 49%-60% And the sum of the mass percentages of the above components is 100%. | Dissolve dithranol in lactic acid to obtain a clear solution.Prepare an aqueous solution of hydroxypropyl methylcellulose, then add and mix in glycerin. While stirring, incorporate the dithranol solution to form the final ointment. | UV-Vis spectrophotometry: Accurately measure 5 ml each of the test solution and the reference solution, transfer each to a 25 ml volumetric flask, accurately add 1 ml of 5% sodium nitrite solution to each, and measure the absorbance of each at a wavelength of 450 nm, then calculate. | Chinese Pharmacopoeia Commission. Chinese Pharmacopoeia 2020 Online Database [DB/OL]. [2025.12.27].  CN103932978B |
|  | Runzao Zhiyang Capsule | Polygonum multiflorum 291g, Processed Polygonum multiflorum 265g, Rehmannia glutinosa 429g, Morus alba leaf 291g, Sophora flavescens 291g, Boehmeria nivea 150g | For the above six herbs: Processed Polygonum multiflorum is pulverized into fine powder and set aside. The remaining five herbs are decocted with water three times, each time for 1 hour. The decoctions are combined, filtered, and concentrated to a thick extract with a relative density of 1.38–1.42 (at 25°C). The powder from the processed Polygonum multiflorum is added, mixed well, dried at 75–80°C, pulverized, and filled into capsules. | TLC: (1) Using benzene-acetone-ethyl acetate-concentrated ammonia solution (2:3:4:0.2) as the developing agent.  (2) Using the upper layer solution of toluene-ethyl acetate-formic acid (5:2:1) as the developing agent. | National Medical Products Administration. |
|  | Compound Zeqi Granules | Euphorbia helioscopia, Hedyotis diffusa, Isatis indigotica leaf, Isatis indigotica root, Spatholobus suberectus vine, Smilax glabra rhizome, Scutellaria barbata, Cyrtomium fortunei, Gentiana scabra, Scutellaria baicalensis, Curcuma phaeocaulis, Schisandra chinensis | / | / | (Dandan 2019) |
|  | Longzhu Ointment | Artificial Moschus, Borax, Calamina (calcined), Sal Ammoniac, Borneolum Syntheticum, Artificial Calculus Bovis, Margarita, Succinum. Excipients include Yellow Vaseline, Lanolin, Liquid Paraffin. | / | The process involves dissolving the sample in chloroform, extracting zinc ions into dilute hydrochloric acid, and then performing a complexometric titration. In the titration, zinc is complexed in an ammoniacal buffer solution, and the endpoint is determined using Eriochrome Black T indicator, titrating with standard disodium edetate (EDTA) solution. The ZnO content is calculated based on the titration volume, where 1 ml of 0.05 mol/L EDTA corresponds to 4.069 mg of ZnO, and the product must contain no less than 5.8% ZnO. | New Drug Conversion Standard. Page: X28-37 Standard Number: WS3-279(Z-043)-2000(Z) |
|  | Calcipotriol Betamethasone Ointment | 1g: Calcipotriol 50μg: Betamethasone 0.5 mg. | Prepared using Caprylic Triglycerides PEG-8 Esters as the base, Calcipotriol and Betamethasone Dipropionate micropowders are uniformly dispersed under high-speed shear homogenization conditions. Leveraging the affinity between this base and common ointment bases, an ointment with a uniform particle size distribution can be obtained. | HPLC :Intersil C18 column, with methanol-acetonitrile-phosphate buffer as the mobile phase, detecting the contents of both Calcipotriol and Betamethasone simultaneously at 265 nm wavelength. | (Shi Yan 2011) |
| **Hospital-prepared Formula** | TCM Decoction | Bambusae Folium, Margaritifera Concha, Glycyrrhizae Radix et Rhizoma each 10g; Sophorae Flavescentis Radix, Dictamni Cortex, Gardeniae Fructus, Houttuyniae Herba, Artemisiae Scopariae Herba each 15g; Scutellariae Radix, Phellodendri Cortex, Gypsum Fibrosum Crudus, Rhei Radix et Rhizoma (to be decocted first), Smilacis Glabrae Rhizoma each 20g. | Decoct in water and take orally in 3 doses, 100 mL each time. | / | (Zhang Hongbo 2019) |
|  | Modified Qingying Decoction | Lonicerae Japonicae Flos 20g; Plantaginis Semen, Imperatae Rhizoma, Spatholobi Caulis, Isatidis Radix each 15g; Rehmanniae Radix Recens, Taraxaci Herba, Moutan Cortex, Forsythiae Fructus, Paeoniae Radix Rubra each 10g. | Decoct in water for oral administration. | / | (Bao Xijing 2016) |
|  | Modified Yangxue Jiedu Decoction: | Plantaginis Semen, Polygoni Multiflori Caulis, Spatholobi Caulis, Isatidis Radix each 15g; Salviae Miltiorrhizae Radix et Rhizoma 12g; Angelicae Sinensis Radix, Taraxaci Herba, Asparagi Radix, Ophiopogonis Radix each 10g. | Decoct in water for oral administration. | / |  |
|  | Modified Qingying Tang | antelope horn powder 0.3 g  Rehmanniae Radix, Moutan Cortex, Paeoniae Radix Rubra, Forsythiae Fructus, Taraxaci Herba：10 g each  Lonicerae Flos 20 g, Imperatae Rhizoma, Isatidis Radix, Spatholobi Caulis, Plantaginis Semen:15 g each | Decoct with water and administer orally. | / | (Li Wei 2011) |
|  | Modified Yangxue Jiedu Tang | Angelicae Sinensis Radix, Asparagi Radix, Ophiopogonis Radix, Taraxaci Herba：10 g each  Polygoni Multiflori Caulis, Isatidis Radix, Spatholobi Caulis, Plantaginis Semen：15 g each  Salviae Miltiorrhizae Radix et Rhizoma：12 g | Decoct with water and administer orally. | / | (Li Wei 2011) |
|  | Chinese Herbal Medicine （cold-dampness obstruction pattern） | Poria cocos 10 g, Scorpio 5 g, Cinnamomum cassia twig 6 g, Atractylodes lancea rhizome 10 g, Spatholobus suberectus vine 15 g, Angelica pubescens root 10 g, processed Aconiti Radix 2 g, Aristolochia debilis stem 10 g, etc. | Decoct with water to 500 ml. | / | (Handing 2014) |
|  | Chinese Herbal Medicine（wind-dampness-toxin-heat pattern） | Paeoniae Radix Rubra, Arnebiae Radix/Lithospermi Radix, Smilacis Glabrae Rhizoma, and Rehmanniae Radix 15 g each, Lonicerae Japonicae Flos 10 g, Chaenomelis Fructus 10 g, Saigae Tataricae Cornu Pulvis 0.6 g, Isatidis Radix 30 g, etc. | Decoct with water to 500 ml. | / | (Handing 2014) |
|  | Chinese Herbal Medicine（blood deficiency-wind dryness and liver-kidney depletion pattern） | Polygoni Multiflori Caulis 30 g, Paeoniae Radix Rubra 15 g, Astragali Radix, Achyranthis Bidentatae Radix, and Angelicae Sinensis Radix 10 g each, Carthami Flos 6 g, Lycii Fructus, Rehmanniae Radix, and Taxilli Herba 15 g each, etc. | Decoct with water to 500 ml. | / | (Handing 2014) |
|  | Self-Prescribed Formula | Rehmanniae Radix, Paridis Rhizoma, Smilacis Glabrae Rhizoma, Sophorae Flos Immaturus, Dictamni Cortex, 15–30 g each; Paeoniae Radix Rubra, Arnebiae Radix/Lithospermi Radix, 10–15 g each. | Decoct to obtain 400 mL. | / | (Liu Zhiyong 2010) |

References

Bao Xijing, Rong Guanghui, Xi Jianyuan, . 2016. "Clinical Observation of 31 Cases of Multiple Plantar Warts Treated with Integrated Chinese and Western Medicine Triple Therapy." *Guiding Journal of Traditional Chinese Medicine and Pharmacy* 22(07):79–80. doi: 10.13862/j.cnki.cn43-1446/r.2016.07.027.

Cheng Xianxiang, Zhou Meng, Ban Xinxin. 2014. "Efficacy Observation of Yanghe Decoction Combined with Acitretin Capsules in Treating 30 Cases of Pustular Psoriasis." *Chinese Journal of Ethnomedicine and Ethnopharmacy* 17:1–1.

Dai Yuntao, Guo Xiaoqing, Zhang Lizeng, Qin Xuemei,. 2007. "Study on Quality Control of Compound Indigo Capsule." *Chinese Traditional Patent Medicine* 29(7):1017–1020.

Dandan, Liu. 2019. "Clinical Efficacy Observation of Modified Yangxue Jiedu Decoction in the Treatment of Psoriasis with Blood-Dryness Pattern and Its Impact on Peripheral Blood IL-22." *Hefei: Anhui University of Chinese Medicine*.

Handing, Jin. 2014. "Analysis of Clinical Efficacy of Integrated Traditional Chinese and Western Medicine in Treating Psoriatic Arthritis." *BETHUNE Medical Journal* 12(4):393–394.

Haobo, Tang. 2012. "Clinical Observation of Cooling Blood and Detoxifying, Benefiting Qi and Strengthening Spleen Method in Treating Psoriasis Vulgaris with Blood-Heat Syndrome." *Yunnan University of Traditional Chinese Medicine*.

Huang Leijuan, Lin Mingsheng，. 2023. "Clinical Observation of Liangxue Jiedu Formula in Treating Acute Radiation Dermatitis." *Yunnan Journal of Traditional Chinese Medicine and Materia Medica* 44(03):67–70.

Jiang Jingyi, Shi Fangchao, Bai Lingling, . 2022. "Study on the Quality Standard of Xiaoyin Granules." *China Pharmaceuticals* 31(05):82–86.

Jiying, Liu. 2022. "Clinical Observation of Modified Jiedu Qingying Decoction in Treating Erythrodermic Psoriasis (Excessive Fire-Toxin Type)." *Heilongjiang Academy of Chinese Medicine Sciences*.

Li Wei, Luo Wei, Cai Ruikang,. 2011. "Comparative study on integrated traditional Chinese and Western medicine in treating erythrodermic psoriasis." *Infection, Inflammation, Repair* 12(3):165–167.

Li Wei, Ma Jia, Wu Jing,. 2014. "Determination of mometasone furoate cream by HPLC." *Anhui Medical and Pharmaceutical Journal* 18(10):1854–1856.

Liu Guihua, Song Yeqiang. 2017. "Modified Longdan Xiegan Decoction Combined with Longzhu Ointment in Treating 30 Cases of Palmoplantar Pustular Psoriasis."*.Henan Traditional Chinese Medicine* 37(4):699–702.

Liu Hongxia, Yao Shangping. 2009. "Efficacy Observation of Integrated Traditional Chinese and Western Medicine in Treating Erythrodermic Psoriasis." *Chinese Journal of Dermatovenereology* 23(11):731–732.

Liu Zhiyong, Wang Jusheng, Wang Ping, . 2010. "Clinical Observation of Liangxue Jiedu Decoction in Treating Psoriasis Vulgaris (Blood-Heat Syndrome)." *World Journal of Integrated Traditional and Western Medicine* 5(4) (317-320).

Qiuping, Wen. 2023. "Clinical Observation of Modified Huoxue San Yu Decoction in Treating Psoriasis with Blood-Stasis Syndrome." *Heilongjiang Academy of Chinese Medical Sciences* 000007.

Shan Ruiping, Bao Yi, Shang Haiping, Shen Minji, Li Fanzhu,. 2022. "Preparation and in vitro transdermal permeation evaluation of tacrolimus ointment." *Chinese Journal of Modern Applied Pharmacy* 39(08):1082–1087.

Shi Yan, Chen Danxia, He Zhigao,. 2011. "HPLC Simultaneous Determination of Two Main Components in Calcipotriol/Betamethasone Ointment." *Chinese Pharmacy* 22(11):1996–1997.

Su Huanhuan, Huang Xiaowu. 2017. "Prescription Optimization and Stability Study of Urea Ointment." *Military Medical Sciences* 41(07):615–617.

Sun Lixin, Xi Jianyuan, Shen Lele,. 2021. "Clinical Observation of Liangxue Xiaobi Pills Combined with Acitretin in Treating Erythrodermic Psoriasis." *Asia-Pacific Traditional Medicine* 17(12):99–100.

Wenqing., Zhao. 2020. "Efficacy of Qingre Liangxue Jiedu Decoction on Psoriasis Vulgaris with Blood-Heat Syndrome and Its Effect on Peripheral Blood Inflammatory Factors." *Modern Journal of Integrated Traditional Chinese and Western Medicine* 29(18):2006–2009.

Wu Sunsi, Cheng Saiyuan, Gao Shangpu, . 2022. "Effect of Xiegan Liangxue Jiedu Formula on Psoriasis Vulgaris with Blood-Heat Syndrome." *China Medical Herald* 19(28):140–143. doi: 10.16254/j.cnki.53-1120/r.2023.03.003.

Xiong Zhihui, Zhou Xiaoqing. 2017. "Research progress in the determination of Tripterygium glycosides components." *World Latest Medicine Information* 17(99):44–45+50.

Xuling, Wu. 2021. "Therapeutic observation of modified Taohong Siwu Decoction combined with herbal medicated bath in treating blood-stasis psoriasis." *Anhui University of Chinese Medicine* 000338.

Yang Chunxin, Liang Jian, Shen Xiong,. "Study on the HPLC fingerprint of Tripterygium glycosides tablets " *Chinese Pharmaceutical Association,*.

Yang Xinxin, Liu Chuanxin, Yan Changxi, . 2020. "Qualitative analysis of chemical constituents in Huoxue Jiedu Decoction by UPLC-Q-TOF/HRMSE " *Drug Evaluation Research* 43(11):2207–2216.

Zhang Hengpo, Chang Haiyan, Cao Bingqing, . 2017. "Clinical observation of tacalcitol ointment in the treatment of pityriasis simplex." *Chinese Journal of Dermatovenereology of Integrated Traditional and Western Medicine*,* 16(4):342–343.

Zhang Hongbo, Chen Jianshi, Zhang Xiaojun,. 2019. "Observation on the Efficacy of TCM Decoction Combined with TCM Lotion in Treating Psoriasis Vulgaris with Blood-Heat Syndrome." *Journal of Liaoning University of Traditional Chinese Medicine* 21(05):198–201. doi: 10.13194/j.issn.1673-842x.2019.05.054.

Zhang Xiaolan, Li Junchao, Xun Xianghong,. 2025. "Effects and mechanism of Huoxue Jiedu Decoction on skin lesions and inflammatory response in psoriasis mice." *Progress in Anatomical Sciences*:1–6.

Zhang Xuebing, Liu Chengxiang, Yuan Xingxing, . 2017. "Therapeutic effect of Taohong Ershao Decoction on blood-stasis psoriasis vulgaris and its influence on serum TH1/TH2 levels." *Chinese Journal of Modern Distance Education of Traditional Chinese Medicine* 15(10):56–57.

Zhongshan Hospital, Fudan University. 2008. "Proceedings of the 2008 Annual Academic Conference of the Chinese Pharmaceutical Association & the 8th Chinese Pharmacist Week. Dept. of Pharmacy."704–710.
