# Supplementary material for: Efficacy and safety of common Chinese herbal medicines in treating psoriasis: a systematic review and meta-analysis
Source: Front Pharmacol. 2026 Feb 20;17:1718564. doi: 10.3389/fphar.2026.1718564 (PMC12964141; doi:10.3389/fphar.2026.1718564)
Supplement: Supplementary file 4 [file Table1.docx]

1. **Details for Drug Type A (Species or botanical drugs covered in a monograph of a national or regional pharmacopoeia)**

**Table S1. Detailed Information of Yujin Yinxie Tablets**

| ITEM NO. | SECTION/TOPIC | |
| --- | --- | --- |
| 1 | Drug name | Yujin Yinxie Tablets |
| 2 | Composition of Traditional Chinese medicine | Radix Gentianae Macrophyllae, Radix Angelicae Sinensis , Rhizoma Acori Tatarinowii , Cortex Phellodendri Amurensis , Rhizoma Cyperi , Radix Curcumae, Rhizoma Curcumae Zedoariae, Realgar , Semen Strychni Powder , Spina Gleditsiae , Semen Persicae , Flos Carthami , Olibanum，30g each, Sal Ammoniac 12g, Natrii Sulfas Exsiccatus , Radix et Rhizoma Rhei 18g each, Eupolyphaga Steleophaga 36g, Indigo Naturalis, Semen Momordicae ，24g each |
| 3 | Species and Family of Herbs (based on Kew MPNS) | 1. Radix Gentianae Macrophyllae: The dried roots of *Gentiana macrophylla* Pall. (family Gentianaceae);. 2. Radix Angelicae Sinensis: The dried roots of *Angelica sinensis* (Oliv.) Diels (family Apiaceae). 3. Rhizoma Acori Tatarinowii: The dried rhizomes of *Acorus tatarinowii* Schott (family Araceae). 4. Cortex Phellodendri Amurensis: The dried bark of *Phellodendron amurense* Rupr. (family Rutaceae). 5. Rhizoma Cyperi: The dried rhizomes of *Curcuma wenyujin* Y. H. Chen et C. Ling (family Zingiberaceae). 6. Radix Curcumae: The dried tuberous roots of *Curcuma longa* L. (family Zingiberaceae). 7. Rhizoma Curcumae Zedoariae: The dried rhizomes of *Curcuma phaeocaulis* Val. (family Zingiberaceae). 8. Semen Persicae: The dried mature seeds of *Prunus persica* (L.) Batsch or *Prunus davidiana* (Carr.) Franch. (family Rosaceae). 9. Flos Carthami: The dried flowers of *Carthamus tinctorius* L. (family Asteraceae). 10. Radix et Rhizoma Rhei：The dried roots and rhizomes of *Rheum palmatum* L., *Rheum tanguticum* Maxim. ex Balf., or *Rheum officinale* Baill. (family Polygonaceae). 11. Eupolyphaga Steleophaga: The dried female insects of *Eupolyphaga sinensis* Walker or *Steleophaga plancyi* (Boleny) (family Corydiidae). 12. Indigo Naturalis: A processed dried powder, mass, or granules prepared from the leaves or stems and leaves of *Baphicacanthus cusia* (Nees) Bremek. (family Acanthaceae), *Polygonum tinctorium* Ait. (family Polygonaceae), or *Isatis indigotica* Fort. (family Brassicaceae). 13. Semen Momordicae: The dried mature seeds of *Momordica cochinchinensis* (Lour.) Spreng. (family Cucurbitaceae). |
| 4 | Description of the extract and extraction process | The above nineteen ingredients are ground into powder and soaked in 70% ethanol as the solvent for 24 hours, then percolated. The other six ingredients, including soapberry thorn, are boiled in water three times, with the first time for 3 hours, the second time for 2 hours, and the third time for 1 hour. The liquid is filtered, and the filtrate is combined and concentrated into a thick paste, which is then mixed evenly with the above thick paste and fine powder. Drying, crushing, sieving, granulation, drying, tablet pressing, sugar coating or film coating, and it is ready. |
| 5 | Preferred/ main methods for extract characterisation/ chemical analysis | TLC:Prepare a reference solution containing 0.5 mg per mL of berberine hydrochloride in methanol. Develop using the upper layer of n-butanol-glacial acetic acid-water (7:1:2) as the mobile phase. Examine under UV light (365 nm). HPLC:Use octadecylsilane bonded silica gel as the stationary phase. The mobile phase consists of acetonitrile-0.01 mol/L sodium heptanesulfonate mixed with 0.02 mol/L potassium dihydrogen phosphate in equal volumes (pH adjusted to 2.8 with 10% phosphoric acid) (21:79). Detection wavelength: 254 nm. |
| 6 | Pharmacopoeial Standard | Chinese Pharmacopoeia (ChP), Volume I (2020 Edition) |
| 7 | Approval Number | National Drug Approval Number: Z61020914 |

1. **Details for Drug Type B (Species or botanical drugs widely used or traded without a monograph in a national or international pharmacopoeia)**

**Table S2. Detailed Information of Cooling Blood and Detoxifying Formula**

| ITEM NO. | SECTION/TOPIC | |
| --- | --- | --- |
| 1 | Drug name | **Cooling Blood and Detoxifying Formula** |
| 2 | Composition of Traditional Chinese medicine | Moutan Cortex, Phellodendri Cortex, Polygoni Cuspidati Rhizoma et Radix, Sanguisorbae Radix each 20 g, add 500 mL water. |
| 3 | Species and Family of Herbs (based on Kew MPNS) | 1. Moutan Cortex: Dried root bark of *Paeonia suffruticosa* Andr. 2. Phellodendri Cortex: Dried bark of *Phellodendron chinense* Schneid., a plant in the Rutaceae family. 3. Polygoni Cuspidati Rhizoma et Radix: Dried mature fruit of *Schisandra chinensis* (Turcz. Baill.), a plant in the Magnoliaceae family. 4. Sanguisorbae Radix: Dried root of *Angelica sinensis* (Oliv. Diels.), a plant in the Apiaceae family. |
| 4 | Description of the extract and extraction process | Bring to a boil over high heat, simmer until reduced to 100 mL, cool for use. |
| 5 | Alternative methods for extract characterisation/chemical analysis | The filler is octadecylsilane-bonded silica gel; the mobile phase is methanol-water (45:55); the detection wavelength is 274 nm. The content of paeonol (C_9_H_10_O_3_) in this product, calculated as dry weight, shall not be less than 1.2%. |

**Table S3. Detailed Information of Draining Liver, Cooling Blood and Detoxifying Formula**

| ITEM NO. | SECTION/TOPIC | |
| --- | --- | --- |
| 1 | Drug name | **Draining Liver, Cooling Blood and Detoxifying Formula** |
| 2 | Composition of Traditional Chinese medicine | Moutan Cortex, Paeoniae Radix Rubra each 9 g; Isatidis Folium, Arnebiae Radix seu Lithospermi Radix each 15 g; Salviae Miltiorrhizae Radix et Rhizoma, Smilacis Glabrae Rhizoma, Bubali Cornu, Rehmanniae Radix Crudus, Hedyotis Diffusae Herba, Imperatae Rhizoma each 30 g. |
| 3 | Species and Family of Herbs (based on Kew MPNS) | 1. Moutan Cortex:Dried root bark of *Paeonia suffruticosa* Andr. 2. Paeoniae Radix Rubr:Dried roots of *Paeonia lactiflora* Pall. or *Paeonia veitchii* Lynch, both belonging to the Ranunculaceae family. 3. Isatidis Folium:Dried leaves of *Isatis indigotica* Fort. 4. Arnebiae Radix:The dried roots of *Arnebia euchroma* (Royle) Johnst. from Xinjiang or *Arnebia* *guttata* Bungefrom Inner Mongolia, both belonging to the Arnebia family. 5. Salviae Miltiorrhizae Radix et Rhizoma:Dried roots and rhizomes of *Salvia miltiorrhiza* Bge. from the family LipidaceaeSmilacis Glabrae Rhizoma. 6. Smilacis Glabrae Rhizoma:Dried rhizome of *Smilax glabra* Roxb. from the Lamiaceae family. 7. Bubali Cornu:The horns of *Bubalus bubalis* Linnaeus, a member of the Bovidae family. 8. Rehmanniae Radix Crudus:Fresh or dried rhizomes of *Rehmannia glutinosa* Libosch. 9. Imperatae Rhizoma:The dried rhizome of *Imperata* *cylindrica* Beauv. var. major (Nees) C. E. Hubb., a plant of the Poaceae family. |
| 4 | Description of the extract and extraction process | Decoct in water and take warm. |
| 5 | Alternative methods for extract characterisation/chemical analysis | The filler is octadecylsilane-bonded silica gel; the mobile phase is methanol-water (45:55); the detection wavelength is 274 nm. The content of paeonol (C_9_H_10_O_3_) in this product, calculated as dry weight, shall not be less than 1.2%. |

**Table S4. Detailed Information of Clearing Heat, Cooling Blood and Detoxifying Decoction**

| ITEM NO. | SECTION/TOPIC | |
| --- | --- | --- |
| 1 | Drug name | **Clearing Heat, Cooling Blood and Detoxifying Decoction** |
| 2 | Composition of Traditional Chinese medicine | Bistortae Rhizoma 9g; Arnebiae Radix seu Lithospermi Radix, Paeoniae Radix Rubra, Dictamni Cortex each 10g; Lonicerae Japonicae Flos 12g; Sophorae Flos Immaturus, Smilacis Glabrae Rhizoma, Imperatae Rhizoma, Rehmanniae Radix Crudus each 25g |
| 3 | Species and Family of Herbs (based on Kew MPNS) | 1. Bistortae Rhizoma:The dried rhizome of the plant *Polygonum bistorta* L., which belongs to the Polygonaceae family. 2. Arnebiae Radix seu Lithospermi Radix:Dried roots of *Arnebia euchroma* (Royle) Johnst. or *Arnebia guttata* Bunge from the family Arnebiaceae. 3. Paeoniae Radix Rubra:Dried roots of *Paeonia lactiflora* Pall. or *Paeonia veitchii* Lynch, both belonging to the Ranunculaceae family. 4. Dictamni Cortex:Dried root bark of *Dictamnus dasycarpus* Turcz., a plant of the Rutaceae family. 5. Lonicerae Japonicae Flos:Dried flower buds or early-blooming flowers of *Lonicera japonica* Thunb., a plant in the Lonicera family. 6. Sophorae Flos Immaturus:Dried flowers and buds of *Sophora japonica* L. 7. Smilacis Glabrae Rhizoma:Dried rhizome of *Smilax glabra* Roxb. from the Lamiaceae familyImperatae Rhizoma:The dried rhizome of *Imperata cylindrica* Beauv. var. major (Nees) C. E. Hubb., a plant of the Poaceae family. 8. Rehmanniae Radix Crudus:Fresh or dried rhizomes of *Rehmannia glutinosa* Libosch |
| 4 | Description of the extract and extraction process | Routine decoction |
| 5 | Alternative methods for extract characterisation/chemical analysis | The silica gel was bonded with octadecylsilane as the filler; acetonitrile was used as mobile phase A, and 0.04% phosphoric acid solution as mobile phase B, with gradient elution performed according to the specifications in the table below; the detection wavelength was 280 nm. The product, calculated as dry weight, must contain no less than 0.030% gallic acid (C_7_H_6_O_5_), no less than 0.15% chlorogenic acid (C_16_H_18_O_9_), and no less than 0.15% epicatechin (C_15_H_14_O_6_). |

**Table S5. Detailed Information of Cooling Blood and Strengthening Spleen Formula**

| ITEM NO. | SECTION/TOPIC | |
| --- | --- | --- |
| 1 | Drug name | **Cooling Blood and Strengthening Spleen Formula** |
| 2 | Composition of Traditional Chinese medicine | Crataegi Fructus, Sophorae Flavescentis Radix each 15g; Rehmanniae Radix Recens, Sophorae Flos Immaturus, Lonicerae Japonicae Flos, Alismatis Rhizoma each 20g; Bubali Cornu, Astragali Radix Crudus, Paeoniae Radix Rubra, Paris Polyphyllae Rhizoma, Dioscoreae Rhizoma, Coicis Semen Crudus each 30g. |
| 3 | Species and Family of Herbs (based on Kew MPNS) | 1. Crataegi Fructus:The dried mature fruit of *Crataegus pinnatifida* Bge. var. major N. E. Br. or hawthorn (*Crataegus pinnatifida* Bge.). 2. Sophorae Flavescentis Radix:Dried root of *Sophora flavescens* Ait. 3. Rehmanniae Radix Recens:Fresh or dried rhizomes of *Rehmannia glutinosa* Libosch. 4. Sophorae Flos Immaturus:Dried flowers and buds of Sophora japonica L. 5. Lonicerae Japonicae Flos:Dried flower buds or early-blooming flowers of *Lonicera japonica* Thunb., a plant in the Lonicera family. 6. Alismatis Rhizoma:Dried rhizome of *Alisma orientale* (Sam. Juzep.) or *Alisma plantago-aquatica* Linn. 7. Bubali Cornu:The horns of *Bubalus bubalis* Linnaeus, a member of the Bovidae family. 8. Astragali Radix Crudus:The dried root of *Astragalus membranaceus* (Fisch.) Bge. var. *mongholicus* (Bge.) Hsiao, also known as membranaceous *Astragalus membranous* Astragalus. 9. Paeoniae Radix Rubra:Dried roots of *Paeonia lactiflora* Pall. or *Paeonia veitchii* Lynch, both belonging to the Ranunculaceae family. 10. Paris Polyphyllae Rhizoma:The dried rhizomes of *Paris polyphylla* Smith *var. yunnanensis* (Franch.) Hand.-Mazz. or *Paris polyphylla* Smith *var. chinensis* (Franch.) Hara, both belonging to the Lamiaceae family. 11. Dioscoreae Rhizoma:Dried rhizome of *Dioscorea opposita* Thunb. 12. Coicis Semen Crudus:Dried mature seed of *Coix lacryma-jobi* L. var. *mayuen* (Roman. Stapf), a plant of the Poaceae family |
| 4 | Description of the extract and extraction process | One dose every two days, 200mL each time |
| 5 | Alternative methods for extract characterisation/chemical analysis | Take approximately 1 g of the fine powder of this product, weigh it accurately, and add 100 mL of water with precision. Soak at room temperature for 4 hours, shaking occasionally, then filter. Accurately measure 25 mL of the filtrate, add 50 mL of water, and 2 drops of phenolphthalein indicator solution. Titrate with sodium hydroxide titration solution (0.1 mol/L) to obtain the final product. Each 1 mL of sodium hydroxide titration solution (0.1 mol/L) is equivalent to 6.404 mg of citric acid (C_6_H_8_O_7_). The product, calculated as a dry weight, shall contain organic acids (expressed as citric acid, C_6_H_8_O_7_) not less than 5.0% |

**Table S6. Detailed Information of Modified Huoxue San Yu Decoction**

| ITEM NO. | SECTION/TOPIC | |
| --- | --- | --- |
| 1 | Drug name | **Modified Huoxue San Yu Decoction** |
| 2 | Composition of Traditional Chinese medicine | Sparganii Rhizoma, Curcumae Rhizoma, Sappan Lignum, Euonymi Ramulus, Aucklandiae Radix, Tribuli Fructus, Scutellariae Radix each 10g; Paeoniae Radix Rubra, Paeoniae Radix Alba, Persicae Semen, Carthami Flos, Saposhnikoviae Radix, Dictamni Cortex, Lonicerae Japonicae Flos, Forsythiae Fructus, Citri Reticulatae Pericarpium each 15g; Coicis Semen Tostum 20g. |
| 3 | Species and Family of Herbs (based on Kew MPNS) | 1. Sparganii Rhizoma:Dried tuber of *Sparganium stoloniferum* Buch.-Ham. 2. Curcumae Rhizoma:Dried rhizome of *Curcuma phaeocaulis* Val., *Curcuma kwangsiensis* S. G. Lee et C. F. Liang, or *Curcuma wenyujin* Y. H. Chen et C. Ling of the family Zingiberaceae. 3. Sappan Lignum:Dried Heartwood of *Caesalpinia sappan* L. 4. Aucklandiae Radix:Dried root of *Aucklandia lappa* Decne. 5. Scutellariae Radix:Dried root of *Scutellaria baicalensis* Georgi, a plant of the Lamiaceae familyPaeoniae Radix Rubra: 6. Paeoniae Radix Alba:Dried roots of *Paeonia lactiflora* Pall. or *Paeonia veitchii* Lynch, both belonging to the Ranunculaceae family. 7. Persicae Semen:The dried mature seeds of *Prunus persica* (L.) Batsch or *Prunus davidiana* (Carr.) Franch., both belonging to the Rosaceae family. 8. Saposhnikoviae Radix: 9. Dictamni Cortex:Dried root bark of *Dictamnus dasycarpus* Turcz., a plant of the Rutaceae family. 10. Lonicerae Japonicae Flos:Dried flower buds or early-blooming flowers of *Lonicera japonica* Thunb., a plant in the Lonicera familyForsythiae Fructus: 11. Citri Reticulatae Pericarpium: Dried mature fruit peel of *Citrus reticulata* Blanco and its cultivated varieties. 12. Coicis Semen Tostum: The dried mature seed of *Coix lacryma-jobi* L. var. mayuen (Roman. Stapf), a plant of the Poaceae family. |
| 4 | Description of the extract and extraction process | Decoct and take warm |
| 5 | Alternative methods for extract characterisation/chemical analysis | The octadecylsilane-bonded silica gel was used as the filler; the mobile phase was methanol-water-phosphoric acid (47:53:0.2); the detection wavelength was 280 nm. The octadecylsilane-bonded silica gel was used as the filler; the mobile phase was methanol-water-phosphoric acid (47:53:0.2); the detection wavelength was 280 nm. |

**Table S7. Detailed Information of Xiao Yin Tang**

| ITEM NO. | SECTION/TOPIC | |
| --- | --- | --- |
| 1 | Drug name | **Xiao Yin Tang** |
| 2 | Composition of Traditional Chinese medicine | Rehmanniae Radix, Imperatae Rhizoma, Paeoniae Radix Rubra：10 parts each；Smilacis Glabrae Rhizoma, Dictamni Cortex, Anemarrhenae Rhizoma12 parts each；Lonicerae Flos，Saposhnikoviae Radix：6 parts each；Salviae Miltiorrhizae Radix et Rhizoma 15 parts,Glycyrrhizae Radix et Rhizoma 5 parts. |
| 3 | Species and Family of Herbs (based on Kew MPNS) | 1. Rehmanniae Radix: Fresh or dried root tubers of *Rehmannia glutinosa Libosch*. 2. Imperatae Rhizoma: The dried rhizomes of *Imperata cylindrica Beauv. var. major（Nees）C. E. Hubb*. 3. Paeoniae Radix Rubra：The dried roots of *Paeonia lactiflora Pall or Paeonia veitchii Lynch*, 4. Smilacis Glabrae Rhizoma: The dried rhizomes of *Smilax glabra Roxb,* 5. Dictamni Cortex: The dried root bark of *Dictamnus dasycarpus Turcz*, 6. Anemarrhenae Rhizoma: The dried rhizome of *Anemarrhena asphodeloides Bge*, 7. Lonicerae Flos: The dried flower buds or the newly opened flowers of *Lonicera japonica Thunb*, 8. Saposhnikoviae Radix: The dried roots of *Saposhnikovia divaricata (Turcz.) Schischk*, 9. Salviae Miltiorrhizae Radix et Rhizoma: The dried roots and rhizomes of *Salvia miltiorrhiza* Bge, 10. Glycyrrhizae Radix: The dried roots and rhizomes of *Glycyrrhiza uralensis Fisch., Glycyrrhiza inflata Bat., or Glycyrrhiza glabra L*. |
| 4 | Description of the extract and extraction process | Combine the crude drugs, decoct twice with water (first decoction 25–30 min, second 10–15 min), mix the two decoctions, and use the combined liquid. |
| 5 | Alternative methods for extract characterisation/chemical analysis | The stationary phase was octadecylsilane-bonded silica gel; the mobile phase was methanol-0.1% phosphoric acid solution (1:99); the detection wavelength was 210 nm. The content of zeyun alcohol (C_15_H_22_O_10_) in raw rehmannia should not be less than 0.20%. |

**Table S8. Detailed Information of Taohong Siwu Decoction**

| ITEM NO. | SECTION/TOPIC | |
| --- | --- | --- |
| 1 | Drug name | **Taohong Siwu Decoction** |
| 2 | Composition of Traditional Chinese medicine | wine-processed Angelicae Sinensis Radix, Rehmanniae Radix Praeparata, stir-fried Paeoniae Radix Alba：10 parts each；Chuanxiong Rhizoma, soaked Persicae Semen：6 parts each；Carthami Flos: 4 parts. |
| 3 | Species and Family of Herbs (based on Kew MPNS) | 1. Wine-processed Angelicae Sinensis Radix: The dried roots of *Angelica sinensis (Oliv.) Diels*, 2. Rehmanniae Radix Praeparata: Processed products of raw rehmannia root, 3. stir-fried Paeoniae Radix Alba: The dried roots of *Paeonia lactiflora Pall*. 4. Chuanxiong Rhizoma: The dried rootstock of *Ligusticum chuanxiong Hort*. 5. soaked Persicae Semen：The dried and mature seeds of *Prunus persica (L.) Batsch or Prunus davidiana (Carr.) Franch.* 6. Carthami Flos: The dried flowers of *Carthamus tinctorius L*. |
| 4 | Description of the extract and extraction process | Place the above crude drugs in a decocting apparatus, soak, then perform first and second decoctions; mix the two decoctions uniformly. |
| 5 | Preferred/ main methods for extract characterisation/ chemical analysis | HPLC: octadecyl silyl-bonded silica gel; A = acetonitrile, B = 0.05–0.3 % phosphoric acid; gradient elution at 0.8–1.2 mL min-1; column temperature 25–35 °C; detection wavelength 210–400 nm. |

**Table S9. Detailed Information of Modified Taohong Siwu Decoction**

| ITEM NO. | SECTION/TOPIC | |
| --- | --- | --- |
| 1 | Drug name | **Modified Taohong Siwu Decoction** |
| 2 | Composition of Traditional Chinese medicine | Persicae Semen, Chuanxiong Rhizoma, Paeoniae Radix Alba, Angelicae Sinensis Radix, Smilacis Glabrae Rhizoma, Smilacis Chinae Rhizoma：10 g each; Carthami Flos, Spatholobi Caulis, Clematidis Radix et Rhizoma, Salviae Miltiorrhizae Radix et Rhizoma: 15 g each. |
| 3 | Species and Family of Herbs (based on Kew MPNS) | 1. Persicae Semen: The dried and mature seeds of *Prunus persica* (L.) Batsch *or Prunus davidiana* (Carr.) Franch. 2. Chuanxiong Rhizoma: The dried rootstock of *Ligusticum chuanxiong* Hort*.* 3. Paeoniae Radix Alba: The dried roots of *Paeonia lactiflora* Pall*.* 4. Angelicae Sinensis Radix: The dried roots of *Angelica sinensis* (Oliv.) Diels 5. Smilacis Glabrae Rhizoma: The dried rhizome of *Smilax glabra Roxb.* 6. Smilacis Chinae Rhizoma: The dried rhizome of *Smilax china L.* 7. Carthami Flos: The dried flowers of *Carthamus tinctorius L.* 8. Spatholobi Caulis: The dried vine stems of the plant *Spatholobus suberectus Dunn* 9. Clematidis Radix et Rhizoma: The dried roots and rhizomes of *Clematis chinensis Osbeck, Clematis hexapetala Pall., or Clematis manshurica Rupr.* 10. Salviae Miltiorrhizae Radix et Rhizoma: The dried roots and rhizomes of *Salvia miltiorrhiza Bge.* |
| 4 | Description of the extract and extraction process | Decoct one bag with water twice daily; combine the two decoctions to obtain 200 mL of final liquid for each administration. |
| 5 | Alternative methods for extract characterization/chemical analysis | Using octadecylsilane bonded silica gel as the stationary phase; using methanol-water (20:80) as the mobile phase; and the detection wavelength set at 210 nm. This product, calculated on a dry basis, shall contain at least 2.0% of amygdalin (C_20_H_27_NO_11)_. |

**Table S10. Detailed Information of Huoxue Jiedu Decoction**

| ITEM NO. | SECTION/TOPIC | |
| --- | --- | --- |
| 1 | Drug name | **Huoxue Jiedu Decoction** |
| 2 | Composition of Traditional Chinese medicine | Smilacis Glabrae Rhizoma: 30 g; Sophorae Tonkinensis Radix, Glycyrrhizae Radix et Rhizoma: 6 g each；Arnebiae/Lithospermi Radix, Rehmanniae Radix, Moutan Cortex, Scrophulariae Radix, Angelicae Sinensis Radix: 10 g each; Hedyotidis Diffusae Herba, Salviae Miltiorrhizae Radix et Rhizoma: 15 g each. |
| 3 | Species and Family of Herbs (based on Kew MPNS) | 1. Moutan Cortex:Dried root bark of *Paeonia suffruticosa* Andr. 2. Smilacis Glabrae Rhizoma:Dried rhizomes of *Smilax glabra Roxb*. 3. Sophorae Tonkinensis Radix:Dried roots and rhizomes of *Sophora tonkinensis* Gagnep. 4. Glycyrrhizae Radix et Rhizoma:Dried roots and rhizomes of *Glycyrrhiza uralensis* Fisch., *Glycyrrhiza inflata* Bat., or *Glycyrrhiza glabra* L. 5. Arnebiae/Lithospermi Radix:Dried roots of *Arnebia euchroma* (Royle) Johnst. or *Arnebia guttata* Bunge. 6. Rehmanniae Radix:Fresh or dried tuberous roots of *Rehmannia glutinosa* Libosch. 7. Moutan Cortex:Dried root bark of *Paeonia suffruticosa* Andr. 8. Scrophulariae Radix:Dried root of *Scrophularia ningpoensis* Hemsl. 9. Angelicae Sinensis Radix:Dried root of *Angelica sinensis*（Oliv.）Diels 10. Hedyotidis Diffusae Herba:Whole herb of *Scleromitrion diffusum* (Willd.) R. J. Wang 11. Salviae Miltiorrhizae Radix et Rhizoma:Dried roots and rhizomes of *Salvia miltiorrhiza* Bge*.* |
| 4 | Description of the extract and extraction process | Place the weighed crude drugs in a round-bottom flask, add 10 volumes of distilled water, soak 30 min, decoct gently for 2 h, and filter; add 8 volumes of water to the residue, decoct again for 2 h, filter, and combine the two filtrates. |
| 5 | Preferred/ main methods for extract characterisation/ chemical analysis | UPLC: Waters ACQUITY UPLC BEH C18 (100 mm × 2.1 mm, 1.8 µm); column temp. 40 °C; mobile phase A = 0.1 % formic acid in water, B = 0.1 % formic acid in acetonitrile; flow 0.3 mL min-1; injection 5 µL. Gradient: 0 min 98 % A; 0–14 min 98→40 % A; 14.0–16.5 min 40→2 % A; 16.5–20.0 min 2 % A. |

**Table S11. Detailed Information of Taohong Ershao Decoction**

| ITEM NO. | SECTION/TOPIC | |
| --- | --- | --- |
| 1 | Drug name | **Taohong Ershao Decoction** |
| 2 | Composition of Traditional Chinese medicine | Persicae Semen, Paeoniae Radix Rubra, Paeoniae Radix Alba, Curcumae Rhizoma, Salviae Miltiorrhizae Radix et Rhizoma, Spatholobi Caulis, Glycyrrhizae Radix et Rhizoma each 15 g ；Carthami Flos, Chuanxiong Rhizomaeach 20 g ；Sparganii Rhizoma, Euonymi Alatus Ramulus, Rumex madaio Mak., Rehmanniae Radix (raw), Sophorae Flos each 10 g. |
| 3 | Species and Family of Herbs (based on Kew MPNS) | 1. Persicae Semen:Dried mature seeds of *Prunus persica* (L.) Batsch or *Prunus davidiana* (Carr.) Franch. 2. Paeoniae Radix Rubra:Dried roots of *Paeonia lactiflora* Pall. or *Paeonia veitchii* Lynch 3. Paeoniae Radix Alba:Dried root of *Paeonia lactiflora* Pall. 4. Curcumae Rhizoma:Dried rhizomes of *Curcuma phaeocaulis* Val., *Curcuma kwangsiensis* S. G. Lee & C. F. Liang, or *Curcuma wenyujin* Y. H. Chen & C. Ling 5. Salviae Miltiorrhizae Radix et Rhizoma:Dried roots and rhizomes of *Salvia miltiorrhiza* Bge. 6. Spatholobi Caulis:Dried vine stems of *Spatholobus suberectus* Dunn 7. Glycyrrhizae Radix et Rhizoma:Dried roots and rhizomes of *Glycyrrhiza uralensis* Fisch., *Glycyrrhiza inflata* Bat., or *Glycyrrhiza glabra* L. 8. Sparganii Rhizoma:Dried tubers of *Sparganium stoloniferum* Buch.-Ham. 9. Euonymi Alatus Ramulus:The winged young branches or branch wings of *Euonymus alatus* (Thunb.) Sieb. 10. Rumex madaio Mak.:Dried roots of *Rumex patientia* L. or *Rumex crispus* L. 11. Rehmanniae Radix (raw):Fresh or dried tuberous roots of *Rehmannia glutinosa* Libosch. 12. Sophorae Flos:Dried flowers and flower buds of *Sophora japonica* L. |
| 4 | Description of the extract and extraction process | Decoct one daily dose with water and concentrate to 150 mL. |
| 5 | Alternative methods for extract characterisation/chemical analysis | Octadecylsilane-bonded silica gel was used as the filler; methanol-water (20:80) was used as the mobile phase; the detection wavelength was set at 210 nm. Calculated on the dried basis of Persicae Semen, the content of amygdalin (C_20_H_27_NO_11_) shall not be less than 2.0%. |

**Table S12. Detailed Information of Modified Jiedu Qingying Decoction**

| ITEM NO. | SECTION/TOPIC | |
| --- | --- | --- |
| 1 | Drug name | **Modified Jiedu Qingying Decoction** |
| 2 | Composition of Traditional Chinese medicine | Taraxacum mongolicum, Smilax glabra rhizome 30g each, Paeonia veitchii root, Lonicera japonica flower, Imperata cylindrica rhizome, Forsythia suspensa, Moutan cortex, Rehmannia glutinosa, Tribulus terrestris 20g each, Saposhnikovia divaricata root, Phaseolus radiatus coat, Rubia cordifolia root, Ligusticum chuanxiong rhizome, Angelica sinensis, Gardenia jasminoides fruit 15g each, Coptis chinensis rhizome 10g. |
| 3 | Species and Family of Herbs (based on Kew MPNS) | 1. Taraxacum mongolicum:Dried whole herbs of *Taraxacum mongolicum* Hand.-Mazz., *Taraxacum borealisinense* Kitam., or several other species of the same genus 2. Smilax glabra rhizome:Dried rhizomes of *Smilax glabra Roxb*. 3. Paeonia veitchii root:Dried roots of *Paeonia veitchii* Lynch 4. Lonicera japonica flower:Dried flower buds or flowers just beginning to bloom of *Lonicera japonica* Thunb. 5. Imperata cylindrica rhizome:Dried rhizome of *Imperata cylindrica* Beauv. var. major (Nees) C. E. Hubb. 6. Forsythia suspensa:Dried fruit of *Forsythia suspensa* (Thunb.) Vahl 7. Moutan cortex:Dried root bark of *Paeonia suffruticosa* Andr. 8. Rehmannia glutinosa:Fresh or dried rhizomes of *Rehmannia glutinosa* Libosch. 9. Tribulus terrestris:Dried mature fruits of *Tribulus terrestris* L. 10. Saposhnikovia divaricata root:Dried root of *Saposhnikovia divaricata* (Turcz.) Schischk. 11. Phaseolus radiatus coat:Seed coat of *Phaseolus radiatus* L. 12. Rubia cordifolia root:Dried roots and rhizomes of *Rubia cordifolia* L. 13. Ligusticum chuanxiong rhizome:Dried rhizome of *Ligusticum chuanxiong* Hort. 14. Angelica sinensis:Dried root of *Angelica sinensis* (Oliv.) Diels 15. Gardenia jasminoides fruit:Dried mature fruits of *Gardenia jasminoides* Ellis 16. Coptis chinensis rhizome:Dried rhizomes of *Coptis chinensis* Franch., *Coptis deltoidea* C. Y. Cheng & Hsiao, or *Coptis teeta* Wall. |
| 4 | Description of the extract and extraction process | Decoct most of the herbs with water multiple times, each time for 1 hours. Combine the decoctions and filter. For herbs containing liposoluble components, ethanol extraction can be used to improve the extraction yield of active components. Concentrate the extract under reduced pressure to remove excess water, obtaining a thick extract. Add ethanol to the concentrated liquid to a certain concentration, let stand for alcohol precipitation, remove impurities, recover ethanol from the supernatant. Subject the purified liquid to low-temperature drying to obtain. |
| 5 | Alternative methods for extract characterisation/chemical analysis | Octadecylsilane-bonded silica gel was used as the filler; methanol was taken as mobile phase A and 0.1% formic acid solution as mobile phase B, with gradient elution performed according to the specified procedure; the detection wavelength was set at 327 nm. Calculated on the dried basis of Taraxacum mongolicum, the content of chicoric acid (C_22_H_18_O_12_) shall not be less than 0.45%. |

**Table S13. Detailed Information of Liangxue Xiaobi Pills**

| ITEM NO. | SECTION/TOPIC | |
| --- | --- | --- |
| 1 | Drug name | **Liangxue Xiaobi Pills** |
| 2 | Composition of Traditional Chinese medicine | Rehmannia glutinosa 15g, Arnebia euchroma/Lithospermum erythrorhizon 10g, Moutan cortex 10g, Salvia miltiorrhiza 15g, Polygonatum sibiricum 15g, Eclipta prostrata 10g, Dioscorea opposita 10g, Hedyotis diffusa 15g, Isatis indigotica leaf 15g, Scutellaria barbata 10g, Dictamnus dasycarpus bark 10g, Glycyrrhiza uralensis 6g. |
| 3 | Species and Family of Herbs (based on Kew MPNS) | 1. Rehmannia glutinosa:Fresh or dried tuberous roots of *Rehmannia glutinosa* Libosch. 2. Arnebia euchroma/Lithospermum erythrorhizon:Dried roots of *Arnebia euchroma* (Royle) Johnst. or *Arnebia guttata* Bunge. 3. Moutan cortex:Dried root bark of *Paeonia suffruticosa* Andr. 4. Salvia miltiorrhiza:Dried roots and rhizomes of *Salvia miltiorrhiza* Bge. 5. Polygonatum sibiricum:Dried rhizomes of *Polygonatum kingianum* Coll. et Hemsl., *Polygonatum sibiricum* Red., or *Polygonatum cyrtonema* Hua 6. Eclipta prostrata:Dried aerial parts of *Eclipta prostrata* L. 7. Dioscorea opposita:Dried rhizome of *Dioscorea opposita* Thunb. 8. Hedyotis diffusa:Whole herb of *Scleromitrion diffusum* (Willd.) R. J. Wang 9. Isatis indigotica leaf:Dried leaves of *Isatis indigotica* Fort. 10. Scutellaria barbata:Dried whole herb of *Scutellaria barbata* D. Don 11. Dictamnus dasycarpus bark:Dried root bark of *Dictamnus dasycarpus* Turcz. 12. Glycyrrhiza uralensis:Dried roots and rhizomes of *Glycyrrhiza uralensis* Fisch., *Glycyrrhiza inflata* Bat., or *Glycyrrhiza glabra* L. |
| 4 | Description of the extract and extraction process | Packaged in aluminum-plastic blister packs, each pill weighs 9g. |
| 5 | Alternative methods for extract characterisation/chemical analysis | Octadecylsilane-bonded silica gel was used as the filler; methanol-0.1% phosphoric acid solution (5:95) was adopted as the mobile phase, and the detection wavelength was set at 203 nm. The content of rehmannioside D (C_27_H_42_O_20_) in Rehmannia glutinosa shall not be less than 0.10%. |

**Table S14. Detailed Information of Qingre Huoxue Formula**

| ITEM NO. | SECTION/TOPIC | |
| --- | --- | --- |
| 1 | Drug name | **Qingre Huoxue Formula** |
| 2 | Composition of Traditional Chinese medicine | Rehmannia glutinosa, Sanguisorba officinalis root, Isatis indigotica leaf, Isatis indigotica root, Spatholobus suberectus vine, Smilax glabra rhizome, Imperata cylindrica rhizome, each 30g; Salvia miltiorrhiza, Moutan cortex, Angelica sinensis, Paeonia lactiflora root (red), Paeonia lactiflora root (white), each 10g; Hedyotis diffusa 15g. |
| 3 | Species and Family of Herbs (based on Kew MPNS) | 1. Rehmannia glutinosa:Fresh or dried tuberous roots of *Rehmannia glutinosa* Libosch. 2. Sanguisorba officinalis root:The dried roots of *Sanguisorba officinalis* L. or *Sanguisorba officinalis* L. var. *longifolia* (Bert.) Yü et Li 3. Isatis indigotica leaf:Dried leaves of *Isatis indigotica* Fort. 4. Isatis indigotica root:Dried root of *Isatis indigotica* Fort. 5. Spatholobus suberectus vine:The dried stems of *Spatholobus suberectus* Dunn 6. Smilax glabra rhizome:Dried rhizomes of *Smilax glabra Roxb*. 7. Imperata cylindrica rhizome:Dried rhizome of *Imperata cylindrica* Beauv. var. major (Nees) C. E. Hubb. 8. Salvia miltiorrhiza:Dried roots and rhizomes of *Salvia miltiorrhiza* Bge. 9. Moutan cortex:Dried root bark of *Paeonia suffruticosa* Andr. 10. Angelica sinensis:Dried root of *Angelica sinensis*（Oliv.）Diels 11. Paeonia lactiflora root (red):Dried roots of *Paeonia lactiflora* Pall. or *Paeonia veitchii* Lynch 12. Paeonia lactiflora root (white):Dried root of *Paeonia lactiflora* Pall 13. Hedyotis diffusa:Whole herb of *Scleromitrion diffusum* (Willd.) R. J. Wang. |
| 4 | Description of the extract and extraction process | Proportion the required herbal materials according to the prescription, with conventional dosages such as Paeonia lactiflora root (red), Moutan cortex, Salvia miltiorrhiza, each 9-15g. Soak the herbs in water for 30 minutes, bring to a boil over high heat, then decoct over low heat for 20-30 minutes. Filter and collect the decoction; the dregs can be decocted a second time. |
| 5 | Alternative methods for extract characterisation/chemical analysis | Octadecylsilane-bonded silica gel was used as the filler; methanol-0.1% phosphoric acid solution (5:95) was adopted as the mobile phase, and the detection wavelength was set at 203 nm. The content of rehmannioside D (C_27_H_42_O_20_) in Rehmannia glutinosa shall not be less than 0.10%. |

**Table S15. Detailed Information of Modified Longdan Xiegan Decoction**

| ITEM NO. | SECTION/TOPIC | |
| --- | --- | --- |
| 1 | Drug name | **Modified Longdan Xiegan Decoction** |
| 2 | Composition of Traditional Chinese medicine | Gentiana scabra, Gardenia jasminoides fruit, Scutellaria baicalensis, Alisma orientale, Angelica sinensis, and Bupleurum chinense, 9g each;Glycyrrhiza uralensis, 6g; Lonicera japonica flower and Smilax glabra rhizome, 30g each; Plantago asiatica seed (wrapped for decoction), Rehmannia glutinosa, and Moutan cortex, 15g each. |
| 3 | Species and Family of Herbs (based on Kew MPNS) | 1. Gentiana scabra: The dried roots and rhizomes of *Gentiana manshurica* Kitag., *Gentiana scabra* Bge., *Gentiana triflora* Pall. or *Gentiana rigescens* Franch. of the Gentianaceae family. 2. Gardenia jasminoides: The dried mature fruits of *Gardenia jasminoides* Ellis of the Rubiaceae family. 3. Scutellaria baicalensis: The dried roots of *Scutellaria baicalensis* Georgi of the Lamiaceae family. 4. Alisma orientale: The dried tubers of *Alisma orientale* (Sam.) Juzep. or *Alisma plantago-aquatica* Linn. of the Alismataceae family. 5. Angelica sinensis: The dried roots of *Angelica sinensis* (Oliv.) Diels of the Apiaceae family. 6. Bupleurum chinense: The dried roots of *Bupleurum chinense* DC. or *Bupleurum scorzonerifolium* Willd. of the Apiaceae family. 7. Glycyrrhiza uralensis: The dried roots and rhizomes of *Glycyrrhiza uralensis* Fisch., *Glycyrrhiza inflata* Bat. or *Glycyrrhiza glabra* L. of the Fabaceae family. 8. Lonicera japonica: The dried flower buds or early-blooming flowers of *Lonicera japonica* Thunb. of the Caprifoliaceae family. 9. Smilax glabra: The dried rhizomes of *Smilax glabra* Roxb. of the Liliaceae family. 10. Plantago asiatica: The dried mature seeds of *Plantago asiatica* L. or *Plantago depressa* Willd. of the Plantaginaceae family. 11. Rehmannia glutinosa: The fresh or dried tubers of *Rehmannia glutinosa* Libosch. of the Scrophulariaceae family. 12. Moutan cortex: The dried root bark of *Paeonia suffruticosa* Andr. of the Ranunculaceae family. |
| 4 | Description of the extract and extraction process | Decoct in water for oral administration. |
| 5 | Alternative methods for extract characterisation/chemical analysis | The stationary phase was octadecylsilane-bonded silica gel; the mobile phase was methanol-water (25:75); the detection wavelength was 270 nm. The content of gentiopicroside (C_16_H_20_O_9_) in Gentiana scabra was not less than 3.0%. |

**Table S16. Detailed Information of Yanghe Decoction**

| ITEM NO. | SECTION/TOPIC | |
| --- | --- | --- |
| 1 | Drug name | **Yanghe Decoction** |
| 2 | Composition of Traditional Chinese medicine | Prepared Rehmannia glutinosa 30g, Cervus nippon antler glue 9g, Cinnamomum cassia bark (superior) 3g, Glycyrrhiza uralensis 3g, Zingiber officinale rhizome (processed) 1.5g, Ephedra sinica 1.5g, Sinapis alba seed 6g |
| 3 | Species and Family of Herbs (based on Kew MPNS) | 1. Rehmannia glutinosa: The fresh or dried root of the plant *Rehmannia glutinosa* Libosch. 2. Cervus nippon antler glue: The ossified antlers of the deer species *Cervus elaphus* Linnaeus or *Cervus nippon* Temminck, or the root base shed in the following spring after the antlers have been stripped of their fur. 3. Cinnamomum cassia bark: The dried bark of the plant C*innamomum cassia* Presl. 4. Glycyrrhiza uralensis: The dried roots and rhizomes of the plants *Glycyrrhiza uralensis* Fisch., *Glycyrrhiza inflata* Bat., or *Glycyrrhiza glabra* L. 5. Zingiber officinale rhizome: The dried root of the plant *Zingiber officinale* Rosc. 6. Ephedra sinica: The dried herbaceous stems of the plants *Ephedra sinica* Stapf, *Ephedra intermedia* Schrenk et C. A. Mey., or *Ephedra equisetina* Bge. 7. Sinapis alba seed: The dried mature seeds of the plant *Sinapis alba* L. or *Brassica juncea* (L.) Czern. et Coss. |
| 4 | Description of the extract and extraction process | Clean and select each herb, accurately weigh each according to the optimal proportions, add 8-20 times the amount of water, soak for 2-12 hours, heat-extract 2-3 times, combine the extracts, collect the volatile oil; let the aqueous extract stand for 12-48 hours, filter and collect the supernatant, concentrate under reduced pressure to obtain an extract or spray dry to obtain dry powder as the aqueous extract. |
| 5 | Alternative methods for extract characterisation/chemical analysis | The stationary phase was octadecylsilane-bonded silica gel; with a mobile phase of methanol-0.1% phosphoric acid solution (5:95), and the detection wavelength set at 203 nm. The content of Dioscin (C_27_H_42_O_20_) should not be less than 0.050%. |

**Table S17. Detailed Information of Compound Indigo Capsule**

| ITEM NO. | SECTION/TOPIC | |
| --- | --- | --- |
| 1 | Drug name | **Compound Indigo Capsule** |
| 2 | Composition of Traditional Chinese medicine | Indigo Naturalis, Rhizoma Cyrtomii, Crataegi Fructus Tostus, Massa Medicata Fermentata (Jian Qu) each 60g; Radix Arnebiae seu Lithospermi, Herba Taraxaci each 80g; Radix et Rhizoma Salviae Militiorrhizae, Rhizoma Dioscoreae Hypoglaucae, Cortex Dictamni, Schisandrae Chinensis Fructus (Alcoholic), Radix Angelicae Dahuricae each 100g; Mume Fructus, Herba Portulacae, Rhizoma Smilacis Glabrae each 200g. |
| 3 | Species and Family of Herbs (based on Kew MPNS) | 1. Indigo Naturalis: The dried powder, lumps or granules obtained by processing the leaves or stems of the plant *Baphicacanthus cusia* (Nees) Bremek., *Polygonum tinctorium* Ait. of the Polygonaceae family, or *Isatis indigotica* Fort. of the Cruciferae family. 2. Rhizoma Cyrtomii: The dried root and leaf stalk remnants of the plant *Dryopteris crassirhizoma* Nakai of the Dryopteridaceae family. 3. Crataegi Fructus Tostus: The dried mature fruits of the plant *Crataegus pinnatifida* Bge. var. *major* N. E. Br. or *Crataegus pinnatifida* Bge. of the Rosaceae family. 4. Radix Arnebiae seu Lithospermi: The dried roots of *Arnebia euchroma* (Royle) Johnst. or *Arnebia guttata* Bunge of the Arnebiaceae family. 5. Herba Taraxaci: The dried mature fruits of the plant *Hippophae rhamnoides* L. of the Chrysanthemum family. 6. Radix et Rhizoma Salviae Militiorrhizae: The dried roots and root stems of *Salvia miltiorrhiza* Bge. of the Lamiaceae family. 7. Rhizoma Dioscoreae Hypoglaucae: The dried root stems of *Dioscorea hypoglauca* Palibin of the Dioscoreaceae family. 8. Cortex Dictamni: The dried root bark of *Dictamnus dasycarpus* Turcz. of the Rutaceae family. 9. Schisandrae Chinensis Fructus: The dried mature fruits of *Schisandra chinensis* (Turcz.) Baill. of the Magnoliaceae family. 10. Cortex Angelicae Dahuricae: The dried roots of *Angelica dahurica* (Fisch. ex Hoffm.) Benth. et Hook. f. or *Angelica dahurica* (Fisch. ex Hoffm.) Benth. et Hook. f. var. *formosana* (Boiss.) Shan et Yuan of the Umbelliferae family. 11. Mume Fructus: The dried near-mature fruits of *Prunus mume* (Sieb.) Sieb. et Zucc. of the Rosaceae family. 12. Portulacae: The dried above-ground parts of *Portulaca oleracea* L. of the Portulacaceae family. 13. Rhizoma Smilacis Glabrae: The dried root stems of *Smilax glabra* Roxb. of the Liliaceae family. |
| 4 | Description of the extract and extraction process | - |
| 5 | Preferred/ main methods for extract characterisation/ chemical analysis | Column: Dalian Elite Hypersil ODS (250mm×4.6mm, 5 μm). Mobile phase: Chloroform-methanol-0.1% acetic acid aqueous solution (6:59:35). Flow rate: 1.0 mL/min. |

**Table S18. Detailed Information of Xiaoyin Granules**

| ITEM NO. | SECTION/TOPIC | |
| --- | --- | --- |
| 1 | Drug name | **Xiaoyin Granules** |
| 2 | Composition of Traditional Chinese medicine | Rehmannia glutinosa, Moutan bark, Paeonia lactiflora (red), Angelica sinensis, Sophora flavescens, Honeysuckle, Scrophularia ningpoensis, Arctium lappa, Cicada slough, Dictamnus dasycarpus, Saposhnikovia divaricata, Isatis indigotica leaf. |
| 3 | Species and Family of Herbs (based on Kew MPNS) | 1. Rehmannia glutinosa: The fresh or dried root of the plant *Rehmannia glutinosa* Libosch. 2. Moutan bark: The dried root bark of the plant *Paeonia suffruticosa* Andr. 3. Paeonia lactiflora (red): The dried root of the plant *Paeonia lactiflora* Pall. or the Chinese red *Paeonia veitchii* Lynch. 4. Angelica sinensis: The dried root of the plant *Angelica sinensis* (Oliv. ) Diels. 5. Sophora flavescens: The dried root of the plant *Sophora flavescens* Ait. 6. Honeysuckle: The dried flower buds or the newly opened flowers of the plant *Lonicera japonica* Thunb. 7. Scrophularia ningpoensis: The dried root of the plant *Scrophularia ningpoensis* Hemsl. 8. Arctium lappa: The dried mature fruit of the plant *Arctium lappa* L. 9. Cicada slough: The exoskeleton shed by the nymph of the black cicada *Cryptotympana atrata* (Fabricius) when it emerges from the cocoon. 10. Dictamnus dasycarpus: The dried root bark of the plant *Dictamnus dasycarpus* Turcz. 11. Saposhnikovia divaricata: The dried root of the plant *Saposhnikovia divaricata* (Turcz. ) Schischk. 12. Isatis indigotica leaf: The dried leaf of the plant *Isatis indigotica* Fort. |
| 4 | Description of the extract and extraction process | Prepared into granules using conventional granule preparation methods. |
| 5 | Preferred/ main methods for extract characterisation/ chemical analysis | HPLC: Lichrospher - C18 (250 mm × 4.6 mm, 5 μm). Mobile phase: Acetonitrile - 0.4% phosphoric acid aqueous solution (10:90, V/V). Flow rate: 1.0 mL/min. Detection wavelength: 327 nm. Column temperature: 30°C. Injection volume: 10 μL. |

**Table S19. Detailed Information of Tripterygium Glycosides Tablets**

| ITEM NO. | SECTION/TOPIC | |
| --- | --- | --- |
| 1 | Drug name | **Tripterygium Glycosides Tablets** |
| 2 | Composition of Traditional Chinese medicine | Tripterygium wilfordii 85–95 parts, traditional Chinese medicine excipient 5–10 parts, magnesium stearate 0.5–5 parts, maltodextrin 5–10 parts.The traditional Chinese medicine excipient is a mixture of Lysimachiae Herba (Jinqiancao), Glycyrrhizae Radix et Rhizoma (Gancao), and Scutellariae Radix (Huangqin) in a mass ratio of (1–3):(1–3):(1–3). |
| 3 | Species and Family of Herbs (based on Kew MPNS) | 1. Tripterygium wilfordii: A plant of the *Tripterygium wilfordii* Hook. f. in the Elaeagnaceae family. 2. Lysimachiae Herba: The dried whole herb of the overland yellow plant *Lysimachia christinae* Hance from the Primulaceae family. 3. Glycyrrhizae Radix et Rhizoma: The dried roots and rhizomes of the plants *Glycyrrhiza uralensis* Fisch., *Glycyrrhiza inflata* Bat., or *Glycyrrhiza glabra* L., which belong to the Leguminosae family. 4. Scutellariae Radix: The dried root of the plant *Scutellaria baicalensis* Georgi from the Lamiaceae family. |
| 4 | Description of the extract and extraction process | First, a blend of Lysimachiae Herba, Glycyrrhizae Radix et Rhizoma, and Scutellariae Radix is pulverized, macerated in ethanol, and subjected to heat-reflux extraction. The resulting filtrate is concentrated under reduced pressure to produce a herbal extract. Next, Tripterygium wilfordii is mixed with neutral alumina, ground, and loaded onto a silica-gel column for purification through sequential soaking and elution with chloroform and a specified organic solvent mixture. The collected eluate is concentrated to obtain solid tripterygium glycosides. Finally, this purified extract is uniformly blended with the herbal extract, magnesium stearate, and maltodextrin, then dried and compressed into tablets. |
| 5 | Preferred/ main methods for extract characterisation/ chemical analysis | TLC: use wilforlide A as the reference standard, cyclohexane–acetone (5:3) as the mobile phase, visualize with 10 % ethanolic sulfuric acid, and heat at 105 °C. HPLC: Diomonisl C18 analytical column; detection wavelength 218 nm; column temperature 35 °C; mobile phase water–acetonitrile; flow rate 1.0 mL min-1; gradient elution. |

**Table S20. Detailed Information of Runzao Zhiyang Capsule**

| ITEM NO. | SECTION/TOPIC | |
| --- | --- | --- |
| 1 | Drug name | **Runzao Zhiyang Capsule** |
| 2 | Composition of Traditional Chinese medicine | Polygonum multiflorum 291g, Processed Polygonum multiflorum 265g, Rehmannia glutinosa 429g, Morus alba leaf 291g, Sophora flavescens 291g, Boehmeria nivea 150g. |
| 3 | Species and Family of Herbs (based on Kew MPNS) | 1. Polygonum multiflorum:This product is the dried tuberous root of the Polygonaceae plant *Polygonum multiflorum Thunb*. It is excavated when the leaves wither in autumn and winter. Both ends are removed, and after washing, larger roots are cut into pieces and dried., 2. Processed Polygonum multiflorum:This product is the processed preparation of *Polygonum multiflorum*. 3. Rehmannia glutinosa: This product is the fresh or dried tuberous root of *Rehmannia glutinosa* Libosch. of the family *Scrophulariaceae*. It is collected in autumn. The root head, fibrous roots, and soil are removed, and it is used fresh; or the root is slowly baked until approximately 80% dry. The former is commonly known as "Fresh Rehmannia Root," while the latter is known as "Dried Rehmannia Root." 4. Morus alba leaf: This product is the dried leaf of *Morus alba L.* of the family *Moraceae*. It is collected after the first frost, with impurities removed, and then dried in the sun. 5. Sophora flavescens: This product is the dried root of *Sophora flavescens Ait*. of the *legume* family. It is excavated in spring and autumn. The root head and small branch roots are removed. After washing, it is dried; or it is cut into slices while fresh and then dried. 6. Boehmeria nivea:This product is the dried root and rhizome of *Boehmeria nivea (L.)* *Gaudich*. of the family *Urticaceae*. It is excavated in winter and spring, washed clean of mud and sand, dried, and then cut into thick slices. |
| 4 | Description of the extract and extraction process | For the above six herbs: Processed Polygonum multiflorum is pulverized into fine powder and set aside. The remaining five herbs are decocted with water three times, each time for 1 hour. The decoctions are combined, filtered, and concentrated to a thick extract with a relative density of 1.38–1.42 (at 25°C). The powder from the processed Polygonum multiflorum is added, mixed well, dried at 75–80°C, pulverized, and filled into capsules. |
| 5 | Alternative methods for extract characterisation/chemical analysis | Using octadecylsilane chemically bonded silica as the filler and methanol-0.1% phosphoric acid (80:20) as the mobile phase, with a detection wavelength of 288 nm. The number of theoretical plates calculated based on the emodin peak should not be less than 5,000. Each pill contains a combined amount of Polygonum multiflorum and processed Polygonum multiflorum equivalent to not less than 0.03 mg of emodin (C_15_H_10_O_5_). |

**Table S21. Detailed Information of Compound Zeqi Granules**

| ITEM NO. | SECTION/TOPIC | |
| --- | --- | --- |
| 1 | Drug name | **Compound Zeqi Granules** |
| 2 | Composition of Traditional Chinese medicine | Euphorbia helioscopia, Hedyotis diffusa, Isatis indigotica leaf, Isatis indigotica root, Spatholobus suberectus vine, Smilax glabra rhizome, Scutellaria barbata, Cyrtomium fortunei, Gentiana scabra, Scutellaria baicalensis, Curcuma phaeocaulis, Schisandra chinensis. |
| 3 | Species and Family of Herbs (based on Kew MPNS) | 1. Isatis indigotica leaf, this product is the dried leaf of *Isatis indigotica* Fort. of the family *Cruciferae*. It is collected in 2-3 batches during summer and autumn, cleaned, and dried in the sun. 2. Isatis indigotica root, this product is the dried root of *Isatis indigotica* Fort. of the family *Cruciferae*. It is excavated in autumn. After removal of soil, it is dried in the sun. 3. Spatholobus suberectus vine, This product is the dried vine stem of *Spatholobus suberectus Dunn* of the family Leguminosae. It is collected in autumn and winter. The branches and leaves are removed. It is then sliced and dried in the sun. 4. Smilax glabra rhizome, this product is the dried rhizome of *Smilax glabra Roxb.* of the Liliaceae family. It is collected in summer and autumn. After removal of the fibrous roots and washing, it is dried; or sliced thinly while fresh and then dried. 5. Scutellaria barbata, this product is the dried herb of *Scutellaria barbata D. Don* of the Lamiaceae family. It is collected in summer and autumn when the stems and leaves are luxuriant, then washed clean and dried in the sun. 6. Cyrtomium fortunei, this product is the dried rhizome and leaf stalk base remnant of *Dryopteris crassirhizoma Nakai* of the family Dryopteridaceae. It is collected in autumn. The leaf stalks and fibrous roots are removed, the soil is cleaned off, and it is then dried in the sun. 7. Gentiana scabra, this product is the dried roots and rhizomes o*f Gentiana manshurica Kitag*., *Gentiana scabra Bge.*, *Gentiana triflora Pall*., or *Gentiana rigescens Franch*. of the family Gentianaceae. The first three species are customarily known as "Longdan" (Gentiana), while the last one is customarily known as "Jianlongdan" (Rigid Gentiana). They are excavated in spring and autumn, washed clean, and dried. 8. Scutellaria baicalensis, this product is the dried root of *Scutellaria baicalensis Georgi* of the Lamiaceae family. It is excavated in spring and autumn. After removal of the fibrous roots and soil, the rough bark is rubbed off following sun-drying, and it is then fully dried in the sun. 9. Curcuma phaeocaulis: This product is the dried rhizome of *Curcuma phaeocaulis Val*., *Curcuma kwangsiensis S. G. Lee et C. F. Liang*, or *Curcuma wenyujin Y. H. Chen et C. Ling* of the family Zingiberaceae. The last one is customarily known as "Wen Ezhu" (Wen Curcuma Rhizome). It is collected in winter after the stems and leaves have withered. After washing, it is steamed or boiled until thoroughly translucent. It is then dried in the sun or at a low temperature, after which the fibrous roots and impurities are removed. 10. Schisandra chinensis:This product is the dried ripe fruit of *Schisandra chinensis (Turcz.) Baill.* of the family Magnoliaceae. It is customarily known as "Bei Wuweizi" (Northern Schisandra Fruit). The fruit is harvested in autumn when ripe, then dried in the sun or steamed before drying. The fruit stalks and impurities are subsequently removed. |
| 4 | Description of the extract and extraction process | - |
| 5 | Alternative methods for extract characterisation/chemical analysis | Using octadecylsilane bonded silica as the stationary phase and methanol-water (75:25) as the mobile phase, with the detection wavelength set at 289 nm. The number of theoretical plates of the column is not less than 4,000, calculated with reference to the indigotin peak.Calculated on the dried basis, the product contains not less than 0.050% of the combined amount of indigo (C_16_H_10_N_2_O_2_) and indigotin (C_16_H_10_N_2_O_2_). |

**Table S22. Detailed Information of Longzhu Ointment**

| ITEM NO. | SECTION/TOPIC | |
| --- | --- | --- |
| 1 | Drug name | **Longzhu Ointment** |
| 2 | Composition of Traditional Chinese medicine | Artificial Moschus, Borax, Calamina (calcined), Sal Ammoniac, Borneolum Syntheticum, Artificial Calculus Bovis, Margarita, Succinum. Excipients include Yellow Vaseline, Lanolin, Liquid Paraffin. |
| 3 | Species and Family of Herbs (based on Kew MPNS) | 1. Margarita, this product is pearls formed by the stimulation of bivalve mollusks such as *Pinctada fucata (Dunker)* of the family Pteriidae, *Hyriopsis cumingii (Lea)* or *Cristaria plicata (Leach)* of the family Unionidae. They are removed from the organism, washed, and dried. 2. Yellow Vaseline, this product is a semi-solid mixture of various hydrocarbons obtained from petroleum. 3. Lanolin, This product is obtained through processing and refining of wool. 4. Liquid Paraffin.This product is a mixture of various liquid hydrocarbons obtained from petroleum. |
| 4 | Description of the extract and extraction process | - |
| 5 | Alternative methods for extract characterisation/chemical analysis | The process involves dissolving the sample in chloroform, extracting zinc ions into dilute hydrochloric acid, and then performing a complexometric titration. In the titration, zinc is complexed in an ammoniacal buffer solution, and the endpoint is determined using Eriochrome Black T indicator, titrating with standard disodium edetate (EDTA) solution. The ZnO content is calculated based on the titration volume, where 1 ml of 0.05 mol/L EDTA corresponds to 4.069 mg of ZnO, and the product must contain no less than 5.8% ZnO. |

\

1. **Details for Drug Type C (Species or botanical drugs derived from plants not widely used or traded)**

**Table S23. Detailed Information of** **TCM Decoction**

| ITEM NO. | SECTION/TOPIC | |
| --- | --- | --- |
| 1 | Drug name | **TCM Decoction** |
| 2 | Composition of Traditional Chinese medicine | *Bambusae Folium, Margaritifera Concha, Glycyrrhizae Radix et Rhizoma* each 10g; *Sophorae Flavescentis Radix, Dictamni Cortex, Gardeniae Fructus, Houttuyniae Herba, Artemisiae Scopariae Herba* each 15g; *Scutellariae Radix, Phellodendri Cortex, Gypsum Fibrosum Crudus, Rhei Radix et Rhizoma* (to be decocted first), *Smilacis Glabrae Rhizoma* each 20g. |
| 3 | Species and Family of Herbs (based on Kew MPNS) | 1. *Bambusae Folium* refers to the dried stems and leaves of the grass species Lophatherum gracile Brongn. 2. *Margaritifera Concha*为refers to the shells of the clam species Hyriopsis cumingii（Lea）、Cristaria plicata（Leach）or the pearl oyster species Pinctada fucata（Dunker）. 3. *Glycyrrhizae Radix et Rhizoma* refer to the dried roots and rhizomes of the leguminous plant Glycyrrhiza uralensis Fisch,Glycyrrhiza inflata Bat,Glycyrrhiza glabra L. 4. *Sophorae Flavescentis Radix*efers to the dried root of the leguminous plant Sophora flavescens Ait. 5. *Dictamni Cortex*refers to the dried root bark of the plan Dictamnus dasycarpus Turcz., which belongs to the Rutaceae family. 6. *Gardeniae Fructus*refers to the dried and mature fruits of the plant Gardenia jasminoides Ellis,which belongs to the Rubiaceae family. 7. *Houttuyniae Herba*refers to the fresh whole herb or dried above-ground parts of the plant Houttuynia cordata Thunb,which belongs to the family Boraginaceae. 8. *Artemisiae Scopariae Herba*refers to the dried aerial parts of the plant Artemisia scoparia Waldst. et Kit. or Artemisia capillaris Thunb.,which belongs to the Asteraceae family. 9. *Scutellariae Radix* refers to the dried roots of the plant Scutellaria baicalensis Georgi,which belongs to the Labiatae family. 10. *Phellodendri Cortex*refers to the dried bark of the plant Phellodendron chinense *Schneid,*which belongs to the Rutaceae family. 11. *Gypsum Fibrosum Crudus*is a sulfate mineral belonging to the gypsum group. It mainly contains hydrated calcium sulfate（CaSO_4_•2H_2_O） 12. *Rhei Radix et Rhizoma* refer to the dried roots and rhizomes of the plants belonging to the Polygonaceae family, namely Rheum palmatum L.,Rheum tanguticum Maxim. ex Balf, Rheum officinale Baill,which are all used for medicinal purposes. 13. *Smilacis Glabrae Rhizoma* refers to the dried rootstock of the plant Smilax glabra *Roxb,*which belongs to the Liliaceae family. |
| 4 | Description of the extract and extraction process | Decoct in water and take orally in 3 doses, 100 mL each time. |
| 5 | Alternative methods for extract characterisation/chemical analysis | *Scutellaria Radix*: The stationary phase is octadecylsilane bonded silica gel; the mobile phase is methanol-water-phosphoric acid (47:53:0.2); the detection wavelength is 280nm. Based on the dry weight, the content of baicalin (C_21_H_18_O_11_) shall not be less than 9.0%. |

**Table S24. Detailed Information of** **Modified Qingying Decoction**

| ITEM NO. | SECTION/TOPIC | |
| --- | --- | --- |
| 1 | Drug name | **Modified Qingying Decoction** |
| 2 | Composition of Traditional Chinese medicine | *Lonicerae Japonicae Flos 20g; Plantaginis Semen, Imperatae Rhizoma, Spatholobi Caulis, Isatidis Radix each 15g; Rehmanniae Radix Recens, Taraxaci Herba, Moutan Cortex, Forsythiae Fructus, Paeoniae Radix Rubra each 10g.* |
| 3 | Species and Family of Herbs (based on Kew MPNS) | 1. *Lonicerae Japonicae Flos* refers to the dried flower buds or the newly opened flowers of the plant Lonicera japonica Thunb, which belongs to the Caprifoliaceae family. 2. *Plantaginis Semen* refers to the dried mature seeds of the plants Plantago asiatica L.or Plantago depressa Willd, both belonging to the Plantaginaceae family. 3. *Imperatae Rhizoma* refers to the dried rootstock of the plant Imperata cylindrica Beauv. var. major（Nees）C. E. Hubb, which belongs to the Poaceae family. 4. *Spatholobi Caulis* refers to the dried vine stem of the plant Spatholobus suberectus Dun, belonging to the Fabaceae family. 5. *Isatidis Radix* is the dried root of the plantIsatis indigotica Fort. 6. *Rehmanniae Radix Recens* is the fresh or dried root of the plant Rehmannia glutinosa *Libosch.* 7. *Taraxaci Herba* refers to the dried whole herb of the plant Taraxacum mongolicum Hand.-Mazz from the Asteraceae family, or the dried herb of the alkali grass Taraxacum borealisinense Kitam or several plants of the same genus. 8. *Moutan Cortex* refers to the dried root bark of the plant *Paeonia suffruticosa Andr*, which belongs to the Ranunculaceae family. 9. *Forsythiae Fructus* is the dried root bark of the plant Forsythia suspensa（Thunb.）Vahl from the Oleaceae family. 10. *Paeoniae Radix Rubra* refers to the dried roots of the plant Paeonia lactiflora Pall or Paeonia veitchii Lynch known as the Sichuan red peony. |
| 4 | Description of the extract and extraction process | Decoct in water for oral administration. |
| 5 | Alternative methods for extract characterisation/chemical analysis | The stationary phase used was octadecylsilane bonded silica gel; the mobile phase A was acetonitrile, and the mobile phase B was 0.1% phosphoric acid solution. The detection wavelength was 327nm. Based on the dry weight, the content of chlorogenic acid (C_16_H_18_O_9_) should not be less than 1.5%, and the total content of phenolic acids, including chlorogenic acid (C_16_H_18_O_9_), 3,5-O-dicaffeoylquinic acid (C_25_H_24_O_12_), and 4,5-O-dicaffeoylquinic acid (C_25_H_24_O_12_), should not be less than 3.8% |

**Table S25. Detailed Information of** **Modified Yangxue Jiedu Decoction**

| ITEM NO. | SECTION/TOPIC | |
| --- | --- | --- |
| 1 | Drug name | **Modified Yangxue Jiedu Decoction** |
| 2 | Composition of Traditional Chinese medicine | *Plantaginis Semen, Polygoni Multiflori Caulis, Spatholobi Caulis, Isatidis Radix* each 15g; *Salviae Miltiorrhizae Radix et Rhizoma* 12g; *Angelicae Sinensis Radix, Taraxaci Herba, Asparagi Radix, Ophiopogonis Radix* each 10g. |
| 3 | Species and Family of Herbs (based on Kew MPNS) | 1. *Plantaginis Semen* refers to the dried and mature seeds of the plant Plantago asiatica L or Plantago depressa Willd of the Plantaginaceae family. 2. *Polygoni Multiflori Caulis* refers to the dried stem of the plant Polygonum multiflorum Thunb of the Polygonaceae family. 3. *Spatholobi Caulis* refers to the dried vine stem of the leguminous plantSpatholobus suberectus Dunn. 4. *Isatidis Radix* is the dried root of the plant Isatis indigotica Fort. 5. *Salviae Miltiorrhizae Radix et Rhizoma* refer to the dried roots and rhizomes of the plant Salvia miltiorrhiza Bge, which belongs to the Labiatae family. 6. *Angelicae Sinensis Radix* refers to the dried roots of the plant An*g*elica sinensis*（Oliv.）Diels, which belongs to the Umbelliferae family.* 7. *Taraxaci Herba* refers to the dried whole plants of the plants Taraxacum mongolicum Hand.-Mazz or Taraxacum borealisinense Kitam several plants of the same genus. 8. *Asparagi Radix* refers to the dried tuberous roots of the plant Asparagus cochinchinensis（Lour.）Merr, which belongs to the Liliaceae family. 9. *Ophiopogonis Radix* refers to the dried tuberous roots of the plant Ophiopogon japonicus（L. f.）Ker-Gawl, which belongs to the Liliaceae family. |
| 4 | Description of the extract and extraction process | Decoct in water for oral administration. |
| 5 | Alternative methods for extract characterisation/chemical analysis | *Ophiopogonis Radix* 2g, crushed, are added to 20 mL of a mixture of trichloromethane and methanol (7:3). They are left to soak for 3 hours, then subjected to ultrasonic treatment for 30 minutes. The mixture is cooled and filtered. The filtrate is evaporated, and the residue is dissolved in 0.5 mL of trichloromethane. This is used as the test sample solution. Another solution of the same preparation is made for the control medicinal material. 6 μL of each of the above solutions is drawn and spot-tested on the same silica gel GF254 thin-layer plate. The plate is developed with a solvent system of toluene-methanol-acetic acid (80:5:0.1), and is examined under a UV lamp (254 nm). |

**Table S26. Detailed Information of** **Modified Qingying Tang**

| ITEM NO. | SECTION/TOPIC | |
| --- | --- | --- |
| 1 | Drug name | **Modified Qingying Tang** |
| 2 | Composition of Traditional Chinese medicine | Saiga tatarica Cornu 0.3g; Rehmanniae Radix, Moutan Cortex, Paeoniae Radix Rubra, Forsythiae Fructus, Taraxaci Herba each 10g; Lonicerae Flos 20g, Imperatae Rhizoma, Isatidis Radix, Spatholobi Caulis, Plantaginis Semen each 15g |
| 3 | Species and Family of Herbs (based on Kew MPNS) | 1. ***Saiga tatarica Cornu* refers to the horns of the animal *Saiga tatarica Linnaeus*, which belongs to the Bovidae family.** 2. ***Rehmanniae Radix* refers to the fresh or dried root of the plant *Rehmannia glutinosa Libosch.*, which belongs to the Scrophulariaceae family.** 3. ***Moutan Cortex* refers to the dried root bark of the plant *Peony suffruticosa Andr*, which belongs to the Ranunculaceae family.** 4. ***Paeoniae Radix Rubra* refers to the dried root of the plants *Paeonia lactiflora Pall* or *Paeonia veitchii Lynch*, which belong to the Ranunculaceae family.** 5. ***Forsythiae Fructus* refers to the dried fruit of the plant *Forsythia suspensa (Thunb.) Vahl*, which belongs to the Oleaceae family.** 6. ***Taraxaci Herba* refers to the dried whole herb of the plants *Taraxacum mongolicum Hand.-Mazz*, *Taraxacum borealisinense Kitam*., or several plants of the same genus, which belong to the Asteraceae family.** 7. ***Lonicerae Flos* refers to the dried flower buds or the newly opened flowers of the plant *Lonicera japonica Thunb*, which belongs to the Caprifoliaceae family.** 8. ***Imperatae Rhizoma* refers to the dried rootstock of the plant *Imperata cylindrica Beauv. var. major (Nees) C. E. Hubb.*, which belongs to the Poaceae family.** 9. ***Isatidis Radix* refers to the dried root of the plant *Isatis indigotica Fort*, which belongs to the Cruciferae family.** 10. ***Spatholobi Caulis* refers to the dried vine stem of the plant *Spatholobus suberectus Dunn*, which belongs to the Fabaceae family.** 11. ***Plantaginis Semen* refers to the dried mature seeds of the plants *Plantago asiatica L.* or *Plantago depressa Willd.,* which belong to the Plantaginaceae family.** |
| 4 | Description of the extract and extraction process | Decoct in water for oral administration. |
| 5 | Alternative methods for extract characterisation/chemical analysis | *Lonicerae Flos:* The stationary phase used is octadecylsilane bonded silica gel; the mobile phase A is acetonitrile, and the mobile phase B is 0.1% phosphoric acid solution. Gradient elution is carried out according to the specifications in the table; the column temperature is not higher than 25℃; the flow rate is 0.7 ml per minute; the detection wavelength is 327 nm. The theoretical plate number should be no less than 10,000 based on the green protoxin peak. Based on the dry sample, the content of lonicoside(C_21_H_20_O_11_) should not be less than 0.050%. |

**Table S27. Detailed Information of** **Modified Yangxue Jiedu Tang**

| ITEM NO. | SECTION/TOPIC | |
| --- | --- | --- |
| 1 | Drug name | **Modified Yangxue Jiedu Tang** |
| 2 | Composition of Traditional Chinese medicine | *Angelicae Sinensis Radix, Asparagi Radix, Ophiopogonis Radix, Taraxaci Herba* each 10g ;*Polygoni Multiflori Caulis, Isatidis Radix, Spatholobi Caulis, Plantaginis Semen* each 15g; *Salviae Miltiorrhizae Radix et Rhizoma* 12g. |
| 3 | Species and Family of Herbs (based on Kew MPNS) | 1. *Angelica sinensis Radix* refers to the dried roots of the plant *Angelica sinensis (Oliv.) Diels,* which belongs to the Apiaceae family. 2. *Asparagi Radix* refers to the dried tubers of the plant *Asparagus cochinchinensis (Lour.) Merr.*, which belongs to the Liliaceae family. 3. *Ophiopogonis Radix* refers to the dried tubers of the plant *Ophiopogon japonicus (L. f.) Ker-Gawl.,* which belongs to the Liliaceae family. 4. *Taraxaci Herba* refers to the dried whole plants of the plants such as *Taraxacum mongolicum Hand.-Mazz., Taraxacum borealisinense Kitam.,* or several plants of the same genus. 5. *Polygoni Multiflori Caulis* refers to the dried stems of the plant *Polygonum multiflorum Thunb.,* which belongs to the Polygonaceae family. 6. *Isatidis Radix* refers to the dried roots of the plant *Isatis indigotica Fort.*, which belongs to the Cruciferae family. 7. *Spatholobi Caulis* refers to the dried stems of the plant *Spatholobus suberectus Dunn.* 8. *Plantaginis Semen* refers to the dried mature seeds of the plants such as *Plantago asiatica L. or Plantago depressa Willd.,* which belong to the Plantaginaceae family. 9. *Salviae Miltiorrhizae Radix* *et Rhizoma* refers to the dried roots and root stems of the plant *Salvia miltiorrhiza Bge.* |
| 4 | Description of the extract and extraction process | Decoct in water for oral administration. |
| 5 | Alternative methods for extract characterisation/chemical analysis | *Asparagi Radix*:Take 1 gram of the powder, add 25 mL of methanol, ultrasonic treatment for 30 minutes, filter, take the filtrate and recover the solvent until dry. The residue is dissolved with 5 mL of water, pass through the pre-treated C18 solid-phase extraction column, elute with 10 ml each of water, 10% methanol, and methanol. Collect the methanol eluate, recover the solvent until dry, dissolve the residue with 1 ml of methanol, and this is the test sample solution. Also, prepare the control medicinal material solution by the same method as the test sample. Take 6 μl of each of the above two solutions, respectively, and spot them on the same silica gel G thin-layer plate to form stripes. Use the lower layer solution of chloroform-methanol-water (13∶7∶2) at 10℃ for development. Spray with 10% ethanol sulfuric acid solution, heat at 105 ℃ until the spots show clear color development, and observe under sunlight and ultraviolet light (365nm). |

**Table S28. Detailed Information of** **Chinese Herbal Medicine (Cold-dampness Obstruction Pattern)**

| ITEM NO. | SECTION/TOPIC | |
| --- | --- | --- |
| 1 | Drug name | **Chinese Herbal Medicine (Cold-dampness Obstruction Pattern)** |
| 2 | Composition of Traditional Chinese medicine | *Poria* 10g, *Scorpio* 5g, ***Cinnamomi Ramulus*** 6g, *Atractylodis Rhizoma* 10g, *Spatholobi Caulis* 15g, *Angelicae Pubescentis Radix* 10g, *Aconiti Radix Cocta* 2g, *Aristolochia debilis stem* 10g, etc. |
| 3 | Species and Family of Herbs (based on Kew MPNS) | 1. *Poria* refers to the dried mycelium of the fungus *Poria cocos (Schw. ) Wolf* from the Polyporaceae family. 2. *Scorpio* refers to the dried body of the animal *Buthus martensii Karsch* from the Scorpionidae family. 3. *Cinnamomi Ramulus* refers to the dried tender branches of the plant *Cinnamomum cassia Presl* from the Lauraceae family. 4. *Atractylodis Rhizoma* refers to the dried rootstock of the plants *Atractylodes lancea (Thunb.) DC.* or *Atractylodes chinensis (DC.) Koidz.* from the Compositae family. 5. *Spatholobi Caulis* refers to the dried vine stem of the plant *Spatholobus suberectus Dunn* from the Leguminosae family. 6. *Angelicae Pubescentis Radix* refers to the dried root of the plant *Angelica pubescens Maxim. f. biserrata Shan et Yuan* from the Umbelliferae family. 7. *Aconiti Radix Cocta* refers to the dried mother root of the plant *Aconitum carmichaelii Debx.* from the Ranunculaceae family. 8. *Aristolochiae Herba* refers to the dried above-ground parts of the plants *Aristolochia debilis Sieb.et Zucc. or Aristolochia contorta Bge.* from the Aristolochiaceae family. |
| 4 | Description of the extract and extraction process | Decoct with water to 500 mL. |
| 5 | Alternative methods for extract characterisation/chemical analysis | *Aconiti Radix Cocta*:The stationary phase used was octadecylsilane bonded silica gel; the mobile phase A was acetonitrile-tetrahydrofuran (25:15), and the mobile phase B was 0.1 mol/L ammonium acetate solution. The detection wavelength was 235 nm. The theoretical plate number should be no less than 2000 based on the peak of p-hydroxybenzoylexicodendron alkaloid. Based on the dry weight, the total content of p-hydroxybenzoylexicodendron alkaloid (C_32_H_45_NO_10_), p-hydroxybenzoylexicodendron isoalkaloid (C_31_H_43_NO_9_), and p-hydroxybenzoylexicodendron new alkaloid (C_31_H_43_NO_10_) should be 0.070% to 0.15%. |

**Table S29. Detailed Information of** **Chinese Herbal Medicine (wind-dampness-toxin-heat pattern)**

| ITEM NO. | SECTION/TOPIC | |
| --- | --- | --- |
| 1 | Drug name | **Chinese Herbal Medicine (wind-dampness-toxin-heat pattern)** |
| 2 | Composition of Traditional Chinese medicine | *Paeoniae Radix Rubra, Arnebiae Radix, Smilacis Glabrae Rhizoma, Rehmanniae Radix* 15g each, *Lonicerae Japonicae Flos, Chaenomelis Fructus* 10g each, *Saigae Tataricae Cornu* 0.6 g, *Isatidis Radix* 30g, etc. |
| 3 | Species and Family of Herbs (based on Kew MPNS) | 1. *Paeoniae Radix Rubra* refers to the dried roots of the plant *Paeonia lactiflora Pall.* or *Paeonia veitchii Lynch,* which belongs to the Ranunculaceae family. 2. *Arnebiae Radix* refers to the dried roots of the plant *Arnebia euchroma (Royle) Johnst.* or *Arnebia guttata Bunge,* which belongs to the Arnebiaceae family. 3. *Smilacis Glabrae Rhizoma* refers to the dried rhizomes of the plant *Smilax glabra Roxb.,* which belongs to the Liliaceae family. 4. *Rehmanniae Radix* refers to the fresh or dried root tubers of the plant *Rehmannia glutinosa Libosch.,* which belongs to the Scrophulariaceae family. 5. *Lonicerae Flos* refers to the dried flower buds or the flowers that are just beginning to bloom of the plant *Lonicera japonica Thunb.,* which belongs to the Caprifoliaceae family. 6. *Chaenomelis Fructus* refers to the dried near-mature fruits of the plant *Chaenomeles speciosa (Sweet) Nakai,* which belongs to the Rosaceae family. 7. *Saigae Tataricae Cornu* refers to the horns of the animal *Saiga tatarica Linnaeus* of the Bovidae family. 8. *Isatidis Radix* refers to the dried roots of the plant *Isatis indigotica Fort.*, which belongs to the Cruciferae family. |
| 4 | Description of the extract and extraction process | Decoct with water to 500 mL. |
| 5 | Alternative methods for extract characterisation/chemical analysis | *Isatidis Radix:*The stationary phase used is octadecylsilane bonded silica gel; the mobile phase is methanol - 0.02% phosphoric acid solution (7:93); the detection wavelength is 245nm. The theoretical plate number should be no less than 5000 based on the (R, S)-gaoyichen peak. Based on the dry weight, the content of (R, S)-gaoyichen (C_5_H_7_NOS) should not be less than 0.020%. |

**Table S30. Detailed Information of** **Chinese Herbal Medicine (blood deficiency-wind dryness and liver-kidney depletion pattern)**

| ITEM NO. | SECTION/TOPIC | |
| --- | --- | --- |
| 1 | Drug name | **Chinese Herbal Medicine (blood deficiency-wind dryness and liver-kidney depletion pattern)** |
| 2 | Composition of Traditional Chinese medicine | *Polygoni Multiflori Caulis* 30g, *Paeoniae Radix Rubra* 15g, *Astragali Radix, Achyranthis Bidentatae Radix, and Angelicae Sinensis Radix* 10g each, *Carthami Flos* 6g, *Lycii Fructus, Rehmanniae Radix, Taxilli Herba* 15g each, etc. |
| 3 | Species and Family of Herbs (based on Kew MPNS) | 1. *Polygoni Multiflori Caulis* is the dried stem of the plant *Polygonum multiflorum Thunb*, which belongs to the Polygonaceae family. 2. *Paeoniae Radix Rubra* is the dried root of the plant Paeonia lactiflora *Pall* or Paeonia veitchii *Lynch,* belonging to the Ranunculaceae family. 3. Astragali Radixis the dried root of the plant Astragalus membranaceus（Fisch.）Bge. var. mongholicus（Bge.) Hsiao or Astragalus membranaceus（Fisch. ) Bge, belonging to the Leguminosae family. 4. *Achyranthis Bidentatae Radix* is the dried root of the plantAchyranthes bidentata Bl, belonging to the Amaranthaceae family. 5. *Angelicae Sinensis Radix* is the dried root of the plant An*g*elica sinensis*（Oliv.）Diels,* belonging to the Umbelliferae family. 6. *Carthami Flos* is the dried flower of the plant Carthamus tinctorius *L,*belonging to the Asteraceae family. 7. *Lycii Fructus* is the dried mature fruit of the plant Lycium barbarum L,belonging to the Solanaceae family. 8. *Rehmanniae Radix* is the fresh or dried root of the plant Rehmannia glutinosa *Libosch,*belonging to the Scrophulariaceae family. 9. *Taxilli Herba* is the dried leafy stem of the plant Taxillus chinensis (DC.) Danser, belonging to the Smilaxaceae family. |
| 4 | Description of the extract and extraction process | Decoct with water to 500 mL. |
| 5 | Alternative methods for extract characterisation/chemical analysis | *Polygoni Multiflori Caulis:*The stationary phase is octadecylsilane bonded silica gel; the mobile phase is acetonitrile-water (26:74)； the detection wavelength is 320 nm. The theoretical plate number， calculated based on the 2,3,5,4′-tetrahydroxydiphenyl ether-2-O-β-D-glucoside peak， should be no less than 2000. Based on the dry sample， the content of 2,3,5,4′-tetrahydroxydiphenyl ether-2-O-β-D-glucoside (C_20_H_22_O_9_) should not be less than 0.20%. |

**Table S31. Detailed Information of** **Cooling Blood and Detoxifying Decoction**

| ITEM NO. | SECTION/TOPIC | |
| --- | --- | --- |
| 1 | Drug name | **Cooling Blood and Detoxifying Decoction** |
| 2 | Composition of Traditional Chinese medicine | *Rehmanniae Radix, Paridis Rhizoma, Smilacis Glabrae Rhizoma, Sophorae Flos, Dictamni Cortex* 15~30g each; *Paeoniae Radix Rubra, Arnebiae Radix* 10~15g each. |
| 3 | Species and Family of Herbs (based on Kew MPNS) | 1. *Rehmanniae Radixrefers* to the fresh or dried tuberous roots of the plant Rehmannia glutinosa *Libosch,which belongs to the family Rehmanniaceae.* 2. *Paridis Rhizoma* refers to the dried rootstock of the plant Paris polyphylla Smith var. yunnanensis（Franch. ）Hand.-Mazz. or the plant seven-leaf monkshood Paris polyphylla Smith var. chinensis（Franch. ）Hara of the Liliaceae family. 3. *Smilacis Glabrae Rhizoma* refers to the dried rootstock of the plant light-leaf sweet potato vine Smilax glabra Roxb of the Liliaceae family. 4. *Sophorae Flos* refers to the dried flowers and flower buds of the leguminous plant Sophora japonica L. 5. *Dictamni Cortex*is the dried root bark of the Rutaceae plant White Sophora Dictamnus dasycarpus Turcz. 6. *Paeoniae Radix Rubra* refers to the dried roots of the plantPaeonia lactiflora Pall or Paeonia veitchii Lynch from the Ranunculaceae family. 7. *Arnebiae Radix* refers to the dried roots of the plant Arnebia euchroma（Royle）Johnst or Arnebia guttata Bunge from the Arnebiaceae family. |
| 4 | Description of the extract and extraction process | Decoct to obtain 400 mL. |
| 5 | Alternative methods for extract characterisation/chemical analysis | Rehmanniae Radix:Using octadecylsilane bonded silica gel as the stationary phase; using a mobile phase of methanol-0.1% phosphoric acid solution (1:99); and the detection wavelength is 210 nm. The theoretical plate number should be no less than 5000 based on the Catalpol peak. The content of Catalpol (C_15_H_22_O_10_) in Rehmannia glutinosa should not be less than 0.20%. |
